# Vaccine-Associated Autoimmunity: From Clinical Signals to Immune Pathways

**DOI:** 10.3390/vaccines13111112

**Published:** 2025-10-30

**Authors:** Mou Peng, Zijun Wang

**Affiliations:** 1 Department of Urology, The Second Xiangya Hospital of Central South University, Changsha 410011, China; 2 Department of Dermatology, Shanghai Ninth People’s Hospital, Shanghai Jiao Tong University School of Medicine, Shanghai 200011, China; 3 Laboratory of Precision Dermatology and Molecular Immunology of the Biomaterials and Regenerative Medicine Institute, Shanghai Ninth People’s Hospital, Shanghai Jiao Tong University School of Medicine, Shanghai 200125, China

**Keywords:** vaccine-induced autoimmunity, molecular mimicry, mRNA vaccines, viral vector vaccines, inactivated viral vaccines, protein subunit vaccines

## Abstract

COVID-19 vaccination has played a pivotal role in mitigating the global health crisis and reducing morbidity and mortality associated with SARS-CoV-2 infection. While its public health benefits are unequivocal, the unprecedented scale of vaccination—reaching billions worldwide—has also enabled the detection of rare autoimmune events, including systemic lupus erythematosus, rheumatoid arthritis, type 1 diabetes, and Guillain–Barré syndrome. Although such events occur in only a small subset of individuals, often influenced by genetic, environmental, or dosage-related factors, they underscore the importance of understanding immune tolerance mechanisms in vaccination. This review synthesizes clinical observations and immunological findings from the COVID-19 vaccination era, highlighting key mechanisms such as molecular mimicry, adjuvant-induced inflammation, bystander activation, epitope spreading, and polyclonal B cell activation. We also consider how novel vaccine platforms, particularly mRNA-based technologies, may influence immune regulation and self-tolerance. Importantly, we discuss the therapeutic management of vaccine-associated autoimmunity, including the use of corticosteroids, intravenous immunoglobulin (IVIG), plasma exchange, disease-modifying anti-rheumatic drugs (DMARDs), and other immunosuppressive agents, many of which have led to favorable clinical outcomes. By integrating mechanistic insights with treatment strategies, this review emphasizes that the overall benefits of COVID-19 vaccination overwhelmingly outweigh the risks, while advocating for continued surveillance, mechanistic research, and risk stratification to inform safer and more targeted vaccination strategies in future pandemics.

## 1. Introduction

Vaccination remains one of the most powerful and cost-effective public health strategies for preventing infectious diseases and reducing global morbidity and mortality. Over the past century, the development and deployment of vaccines have led to the successful control or eradication of multiple pathogens, including smallpox, poliovirus, and measles. Continuous advances in vaccine technology—ranging from live attenuated and inactivated vaccines to subunit, viral vector, and, more recently, mRNA-based platforms—have significantly expanded the scope of vaccine-preventable diseases and accelerated responses to emerging pathogens.

**Figure 1:**
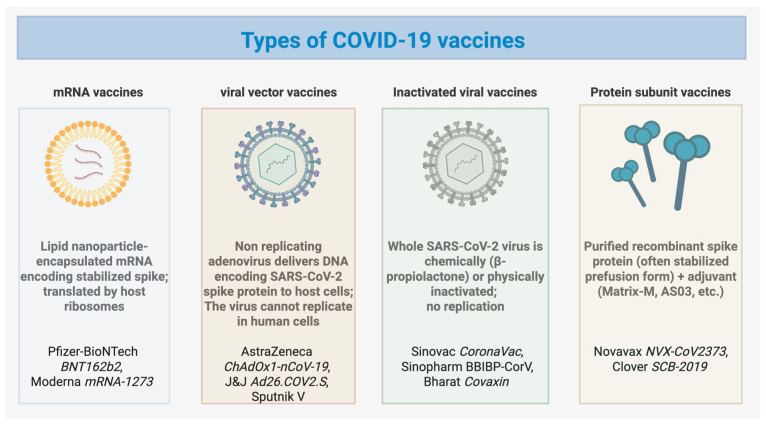
Types of COVID-19 vaccines. Overview of the four main COVID-19 vaccine platforms. mRNA vaccines (e.g., *Pfizer–BioNTech BNT162b2*, *Moderna mRNA-1273*) use lipid nanoparticle–encapsulated mRNA encoding the stabilized spike protein, translated by host ribosomes. Viral vector vaccines (e.g., *AstraZeneca ChAdOx1-nCoV-19*, *J&J Ad26.COV2.S*, *Sputnik V*) employ non-replicating adenoviruses carrying DNA that encodes the spike protein. Inactivated viral vaccines (e.g., *Sinovac CoronaVac*, *Sinopharm BBIBP-CorV*, *Bharat Covaxin*) contain chemically or physically inactivated whole SARS-CoV-2 virions that cannot replicate. Protein subunit vaccines (e.g., *Novavax NVX-CoV2373*, *Clover SCB-2019*) consist of purified recombinant spike proteins—often stabilized in the prefusion form—combined with adjuvants such as Matrix-M or AS03 to enhance immunogenicity.

The emergence of coronavirus disease 2019 (COVID-19), caused by severe acute respiratory syndrome coronavirus 2 (SARS-CoV-2), was first reported in Wuhan, China, in late 2019 [1], leading to a rapid global spread. The rapid development and deployment of COVID-19 vaccines have been pivotal in controlling viral transmission and dramatically reducing severe disease, hospitalization, and mortality [2]. Researchers rapidly developed multiple vaccines using different vector technologies to combat the virus. Notably, seven of these vaccines (Pfizer-BioNTech COVID-19 Vaccine (Comirnaty)—an mRNA vaccine; Moderna COVID-19 Vaccine (Spikevax)—an mRNA vaccine; AstraZeneca/Oxford COVID-19 Vaccine (Vaxzevria, Covishield)—a viral vector vaccine, Johnson & Johnson’s Janssen COVID-19 Vaccine—a viral vector vaccine; Sinopharm COVID-19 Vaccine (BBIBP-CorV)—an inactivated virus vaccine; Sinovac-CoronaVac—an inactivated virus vaccine; Novavax COVID-19 Vaccine (Nuvaxovid, Covovax)—a protein subunit vaccine) have received emergency use approval from the World Health Organization (WHO) (Figure 1). By mimicking the entry process of the virus, vaccines prompt the immune system to generate a robust response, enabling swift recognition and neutralization upon encountering the actual virus [3]. The fundamental mechanism underlying vaccines involves priming the immune system to recognize and mount a defense against the virus, thereby preventing infection. Consequently, the primary objective of vaccination is pre-emptive, aimed at thwarting infection rather than treating established cases.

Despite the overwhelming benefits of COVID-19 vaccination in curbing severe disease and saving millions of lives, rare autoimmune phenomena have been reported in temporal association with immunization. These include conditions such as systemic lupus erythematosus, rheumatoid arthritis, type 1 diabetes, autoimmune hepatitis, and Guillain–Barré syndrome [4]. Importantly, large pharmacovigilance and cohort studies indicate that the incidence of vaccine-associated autoimmune events is extremely low, comparable to or below the baseline rates in unvaccinated populations [5,6,7]. Nonetheless, these observations have prompted investigations into potential mechanisms that might trigger autoimmunity in genetically, environmentally, or dosage-susceptible individuals.

Vaccines can, in rare circumstances, disrupt immune tolerance through processes such as molecular mimicry, bystander activation, epitope spreading, or adjuvant-induced immune stimulation [8,9,10,11]. For COVID-19 vaccines, similarities between the SARS-CoV-2 spike protein and certain human proteins have raised the possibility of molecular mimicry contributing to cross-reactive immune responses [12,13]. Moreover, the potent activation of innate sensors and cytokine cascades—especially type I interferon signaling—may further amplify autoreactive responses in predisposed hosts [14]. Clinically, affected individuals have presented with diverse symptoms ranging from fatigue, arthralgia, and rash to more severe neurological manifestations. However, the causal relationship between vaccination and autoimmunity remains unproven, emphasizing the need for cautious interpretation and rigorous mechanistic validation.

In this review, we provide a balanced and comprehensive overview of autoimmune events reported in the context of COVID-19 vaccination. We aim to integrate clinical observations with mechanistic immunology, elucidating how vaccine components, host immune pathways, and susceptibility factors interact to shape autoimmune risk. Finally, we highlight that, despite rare immune-mediated complications, the overall benefits of COVID-19 vaccination overwhelmingly outweigh potential risks, reinforcing vaccination as a cornerstone of pandemic control and future global health preparedness.

## 2. Induction of Autoimmune Phenomena by COVID-19 Vaccination

Since the initiation of large-scale COVID-19 vaccination programs, retrospective studies and case series have documented cases of new-onset autoimmune diseases post-vaccination. This section includes systemic lupus erythematosus (SLE), rheumatoid arthritis (RA), vaccine-induced immune thrombotic thrombocytopenia (VITT), Guillain–Barré syndrome (GBS), autoimmune hepatitis (AIH), type 1 diabetes mellitus (T1DM), myasthenia gravis (MG), alopecia areata (AA), antiphospholipid syndrome (APS), and ANCA-associated vasculitis (AAV). These diseases are considered critical for investigation to explore potential links with COVID-19 vaccines and to guide the development of therapeutic strategies to manage these conditions if they arise following vaccination (Table 1).

### 2.1. Systemic Lupus Erythematosus (SLE)

Systemic lupus erythematosus (SLE) is a chronic, multisystem autoimmune disorder characterized by the production of autoantibodies that can lead to inflammation and damage in various organs, including the skin, joints, kidneys, heart, lungs, blood vessels, and brain [15,16]. SLE is distinguished by a breakdown in immune tolerance, persistent generation of autoantibodies, heightened activity of B and T lymphocytes, and a loss of immune recognition for self-antigens [17]. The etiology of SLE is multifaceted, involving genetic, environmental, and hormonal factors that contribute to the dysregulation of the immune system [17]. However, the effects of COVID-19 vaccination on the onset and pathogenesis of SLE remain unclear.

Table S1 lists some of the recent new-onset SLE patients after vaccination [18,19,20,21,22,23,24,25,26,27,28,29,30,31,32,33,34]. The age range of these cases varied significantly (14 to 68 years), and initial symptoms differed. However, serological features were generally similar, showing elevated ANA titers, positive anti-dsDNA antibodies, and low complement levels. For example, a 22-year-old female developed right knee pain and fever 15 days post-vaccination, followed by polyarthritis, bilateral foot edema, fingertip rash, and lower limb purpura 10 days later. Laboratory tests revealed positive ANA and elevated immunoglobulin levels. She was diagnosed with SLE and anemia of chronic disease and responded well to prednisone and hydroxychloroquine treatment [18]. Lemoine et al. reported a 68-year-old woman who developed muscle weakness, stiffness, and pain in her extremities after receiving the first dose of the Pfizer-BioNTech COVID-19 vaccine. She initially improved with prednisone treatment, but experienced a recurrence of symptoms two months later. Further testing confirmed a diagnosis of SLE. The patient was treated with methotrexate and prednisone, opting not to receive the second dose of the mRNA vaccine [19]. While the majority of evidence supports the safety and efficacy of COVID-19 vaccines in the general population, including those with autoimmune diseases, some case reports raise questions about the potential for rare adverse events. For instance, Moriyama et al. described a 53-year-old male who developed peritonitis following the administration of the third dose of a SARS-CoV-2 mRNA vaccine and was subsequently diagnosed with SLE [20].

Lupus nephritis (LN) is one of the most severe organ complications of SLE and is a form of glomerulonephritis categorized histologically into six different classes [35]. Instances involving lupus nephritis are exceedingly rare. Data from Table S1 reveals only four reported cases of new-onset biopsy-proven lupus nephritis. Of these, two patients received the AstraZeneca vaccine [31,32], and two received the Pfizer vaccine [33,34]. Specifically, one study [31] noted the onset of class V lupus nephritis within a week of the first AstraZeneca dose, while another [32] documented class III lupus nephritis following the same vaccine. In the Pfizer vaccine group, one pediatric case [33] involved new-onset class V lupus nephritis after the third dose, and another case [34] described a patient who developed new-onset lupus nephritis after the first dose of the Pfizer COVID-19 vaccine. All these patients received immunosuppressive therapy, achieving varying degrees of remission.

Although there is no conclusive evidence to elucidate the relationship between SLE and COVID-19 vaccines, some potential mechanisms include molecular mimicry, type I interferon production, and inflammation mediated by toll-like receptor (TLR) stimulation and vaccine adjuvants triggering autoimmune events [28]. These findings suggest that COVID-19 vaccination might trigger SLE in susceptible individuals, warranting further investigation into this association.

### 2.2. Rheumatoid Arthritis (RA)

Rheumatoid arthritis (RA) is a symmetrical polyarticular condition that primarily affects the small diarthrodial joints of the hands and feet. Beyond synovial inflammation, the aggressive growth of pannus tissue contributes to the destruction of local articular structures. In RA, the synovium, typically a noncellular tissue, becomes infiltrated by CD4^+^ T cells, B cells, and macrophages, leading to inflammation. Rheumatoid factors (RF) and other autoantibodies (e.g., anti-citrullinated protein antibodies (ACPA)) can form immune complexes that activate complement and release chemotactic factors like C5a, drawing inflammatory cells to the joint and exacerbating local damage. Neutrophils in the synovial fluid play a significant role by ingesting these immune complexes and releasing proteolytic enzymes [36,37,38]. Table S2 recorded that eight cases of newly developed RA following COVID-19 vaccination have been reported, with a higher incidence among females [25,39,40,41,42,43,44,45]. Symptoms such as inflammatory joint pain and stiffness appeared within a few days to weeks post-vaccination. Serological testing confirmed RA through the presence of rheumatoid factor and anti-citrullinated protein antibodies. Among these cases, six were associated with mRNA vaccines, one with a viral vector vaccine, and one with an inactivated vaccine (Table S2). Yonezawa et al. reported the first case of seropositive RA following COVID-19 mRNA vaccination, in which a 54-year-old man with a history of CPFE (combined pulmonary fibrosis and emphysema) developed joint pain after receiving the second dose of the Pfizer vaccine. One day after vaccination, he experienced swelling and pain in his joints that did not resolve. He was referred to the hospital 107 days after vaccination due to persistent joint pain. On admission, he showed signs of polyarthritis with swelling in 26/28 joints and tenderness in 28/28. Blood tests revealed elevated RF and ACPA, leading to a diagnosis of RA. He was treated with methylprednisolone and iguratimod, showing a good clinical response [39]. The onset of the condition typically occurs suddenly, necessitating immediate intervention with corticosteroids, disease-modifying antirheumatic drugs (DMARDs), or biologic therapies to effectively manage and suppress the disease activity. Most patients responded well to treatment, achieving varying degrees of clinical remission. However, Nahra et al. reported symptom recurrence in a patient after tapering prednisone to less than 10 mg daily [40]. In addition, an 88-year-old woman with a history of chronic eosinophilic pneumonia (CEP) developed bilateral joint pain and morning stiffness two months before hospital admission. Ten days prior, she received the COVID-19 vaccine and three days later, experienced fever, dyspnea, cough, and malaise. Chest CT showed lung abnormalities similar to her previous CEP. Tests revealed elevated RF and eosinophil count, and ultrasonography confirmed synovitis, leading to a diagnosis of RA. She was treated with steroids for her respiratory issues and RA, improving after treatment. She later received the second COVID-19 vaccine dose with no complications [41].

The proposed mechanisms for the onset of RA following COVID-19 vaccination encompass several immunological processes. These include vaccine-induced immune activation, where the vaccine stimulates the immune system, potentially leading to an autoimmune response. Molecular mimicry is another key mechanism, where similarities between vaccine components and self-antigens provoke an immune response against the body’s own tissues. Furthermore, the unmasking of a pre-existing autoimmune predisposition can occur, where the vaccine triggers an autoimmune condition that was previously latent. The production of proinflammatory cytokines, such as interleukin-6, tumor necrosis factor-alpha, and type I interferons, is central to the pathogenesis of these autoimmune responses. These cytokines can amplify inflammation and contribute to the tissue damage characteristic of RA [41]. Additionally, the polymer polyethylene glycol, which stabilizes the vaccine’s lipid nanoparticles, could trigger delayed antibody-mediated reactions [10,46]. Toll-like receptors (TLRs), which recognize RNA from the vaccine, can also stimulate the production of proinflammatory mediators and type I interferons, further contributing to the development of autoimmune reactions [44]. It is important to note that these case reports represent a small number of individuals and do not necessarily indicate a causal relationship between COVID-19 vaccination and the development of RA. Further research is needed to better understand the potential risks and mechanisms of vaccine-induced autoimmune phenomena and to develop strategies to mitigate potential adverse effects.

### 2.3. Vaccine-Induced Immune Thrombotic Thrombocytopenia (VITT)

Vaccine-induced immune thrombotic thrombocytopenia (VITT) is an autoimmune condition characterized by antibodies that activate platelets directly, leading to thrombosis affecting both arterial and venous circulation [47]. Among the adverse effects associated with COVID-19 vaccination, thrombocytopenia and thrombosis pose significant risks, particularly in recipients of adenoviral vector vaccines. Affected individuals are often young and previously healthy. Reports from research groups in Norway, Germany, and the United Kingdom have described cases of consumptive coagulopathy in patients following adenoviral vector vaccination, presenting with thrombocytopenia, hypofibrinogenemia, and elevated D-dimer levels, along with incidents of cerebral venous sinus thrombosis associated with anti-platelet factor 4 (PF4) IgG antibodies [48,49,50]. The emergence of VITT as a serious concern following COVID-19 vaccination, especially with adenoviral vector vaccines like the ChAdOx1 nCoV-19 vaccine, underscores the need for vigilance and further research into its mechanisms [51].

Table S3 summarizes various case reports and studies on VITT following COVID-19 vaccination [52,53,54,55,56,57,58,59,60,61,62,63,64,65,66,67,68,69,70,71,72,73,74,75]. Patients’ demographic profiles varied widely, spanning ages from 18 to 86 years, with a notable predominance among females. Symptom onset occurred at diverse intervals post-vaccination, typically ranging from several days to weeks. While most VITT cases were linked to adenoviral vector vaccines, mRNA and inactivated vaccines also contributed in some instances. Key laboratory findings consistently included elevated D-dimer levels, reduced platelet counts, and the presence of anti-PF4 antibodies, which confirm the diagnosis of VITT. The persistence and clinical implications of pathogenic anti-PF4 antibodies in VITT remain poorly understood. While many patients show a decline in these antibodies over a median follow-up of 12 weeks [76], some cases exhibit prolonged antibody presence despite treatment efforts. For instance, a 69-year-old patient had detectable platelet-activating anti-PF4 antibodies for over 9 months, despite repeated high-dose intravenous immunoglobulin (IVIG) and therapeutic plasma exchange [54]. Similarly, one case described as “long VITT” involved thrombocytopenia and persistent positive platelet-activation tests lasting over 18 months [55]. The patient continues to suffer from chronic ischemic neuropathy, with uncertainty regarding the cause of ongoing thrombocytopenia—whether due to persistent anti-PF4 antibodies, underlying conditions, or lifestyle factors like alcohol consumption [56]. This highlights the challenges in managing such cases. In addition, a 54-year-old man experienced persistent platelet activation at 3 months post-diagnosis, leading to severe complications and death despite aggressive treatment [57]. The above cases suggest that management of VITT remains challenging, necessitating further research to define optimal care strategies for long-term VITT management, including criteria for diagnosis based on prolonged antibody presence and clinical symptoms. The outcomes of these cases were mixed, with recoveries and fatalities reported, highlighting a significant mortality rate in certain studies, such as Oliveira et al. [58], which documented a 51% mortality rate among 39 cases.

Management strategies for VITT have included anticoagulation, high-dose IVIG, therapeutic plasma exchange, corticosteroids, rituximab, and eculizumab (Table S3). Monitoring of platelet counts, fibrinogen levels, and D-dimer levels in outpatient settings is crucial to assess the effectiveness of treatments and manage potential hypercoagulability. Some patients may develop refractory antibody-induced hypercoagulability, termed “long VITT,” which persists despite treatment efforts with high-dose IVIG and/or anticoagulation.

Greinacher and colleagues [48] believe that COVID-19 vaccination leads to a rare VITT mediated by the activation of PF4 antibodies, which stimulate platelets through Fcγ receptors. This rare side effect is very similar to the clinical presentation of a typical immune-mediated HIT disease that occurs after exposure to heparin. Surprisingly, none of these patients had been exposed to heparin before the onset of the disease. Notably, Huynh et al. identified a key mechanism that showed that the VITT antibody could mimic the effects of heparin by binding to a similar site on PF4 [77]. This allowed PF4 tetramers to aggregate and form immune complexes, which in turn led to Fcγ receptor IIa (FcγRIIa; also known as CD32a)-dependent platelet activation. These results explain how VITT antibody-induced platelet activation could lead to thrombosis. Wang et al.’s research indicates that the disease originally classified as VITT is fundamentally similar to diseases occasionally triggered by adenovirus infections. Consequently, the clinical insights gained from VITT remain pertinent: thrombosis associated with thrombocytopenia and significantly elevated D-dimer levels, particularly following viral infection, should be investigated and managed as anti-PF4 disease. This finding also reveals that the adenovirus itself, rather than other vaccine components, directly or indirectly induces the formation of platelet-activating anti-PF4 antibodies. This discovery holds significant clinical implications for vaccine development, emphasizing the critical importance of identifying the antigen [78].

Considering the reported fatalities linked to ChAdOx1 nCoV-19 VITT post-vaccination, prompt diagnosis and timely therapeutic intervention are crucial for enhancing patient outcomes. Identifying the mechanism by which adenovirus vector COVID-19 vaccines, such as AstraZeneca, induce VITT is essential for addressing and preventing similar side effects in all adenovirus vaccines, as well as for developing effective treatments.

### 2.4. Guillain–Barré Syndrome (GBS)

Guillain–Barré syndrome (GBS) is a rare but severe autoimmune disorder characterized by rapid onset of muscle weakness and paralysis, primarily affecting the peripheral nervous system. The condition is often triggered by infections, with Campylobacter jejuni being a common antecedent, and can also be associated with vaccinations, though the risk is relatively low [79,80,81]. The U.S. Centers for Disease Control and Prevention (CDC) has noted that following the administration of 12.8 million doses of the Johnson & Johnson COVID-19 vaccine, there have been roughly 100 initial reports of GBS. These instances mostly emerged within two weeks of vaccination and were more common among men aged 50 and above (https://www.cdc.gov/vaccine-safety/about/guillain-barre.html?CDC_AAref_Val=https://www.cdc.gov/vaccinesafety/concerns/guillain-barre-syndrome.html (accessed on 26 October 2025)).

Table S4 [82,83,84,85,86,87,88,89,90,91,92,93,94,95,96,97,98,99,100,101,102,103,104,105,106,107,108,109,110,111,112,113,114,115,116,117,118,119,120,121,122,123,124,125,126,127,128,129,130,131,132,133,134,135,136,137,138,139,140,141,142,143,144,145,146,147,148,149,150,151,152,153,154,155,156,157,158,159,160,161,162,163,164,165] recorded 142 cases of GBS. McKean and Chircop reported the case of a 48-year-old man with dyslipidemia who developed GBS after receiving the first dose of the COVID-19 vaccine (Vaxzevria) [82]. A case series [83] presents two case reports of GBS following vaccination with the BNT162b2 mRNA vaccine in elderly women who were in complete remission from diffuse large B-cell lymphoma. The first patient, an 80-year-old, developed progressive weakness and tingling, leading to quadriparesis, and was diagnosed with the acute motor and sensory axonal neuropathy form of GBS, responding to IVIG treatment. The second patient, a 76-year-old, experienced tingling and weakness, diagnosed with the acute inflammatory demyelinating polyneuropathy subtype of GBS, also treated successfully with IVIG. Both cases raise concerns about a potential link between the vaccine and GBS, particularly in patients with underlying immunodeficient.

Although many patients experience improvement in their condition, with some fully recovering after treatment, a few cases report deterioration, necessitating more intensive interventions like mechanical ventilation [84,85,86]. Hasan et al. [84] discussed a 62-year-old female who developed GBS shortly after receiving the Oxford/AstraZeneca COVID-19 vaccine. Within 11 days post-vaccination, she presented with progressive weakness and paraesthesia in her lower limbs. Despite no recent infections, clinical exams, lumbar puncture, and nerve conduction studies confirmed GBS. Treatment included intravenous immunoglobulin, but her condition deteriorated, requiring intensive care due to respiratory involvement and sepsis. In rare instances, patients have died, highlighting the severe nature of GBS in some cases. One case, reported by García-Grimshaw et al. [85], involved a 67-year-old female who developed quadriparesis, loss of deep tendon reflexes, and respiratory failure 4 days after receiving the first dose of the BNT162b2 mRNA vaccine. Seventeen days after hospitalization, the patient died, which was attributed to ventilator-associated pneumonia complicated with septic shock. In another case [86], an 87-year-old male presented with bilateral facial weakness 17 days after receiving the first dose of the Sputnik V vaccine. He experienced distal paresthesias in all four limbs but reported no pain. Physical examination revealed involvement of the lower cranial nerves and decreased deep tendon reflexes in all four limbs. The patient was diagnosed with a variant of GBS known as Bilateral Facial Palsy with Paresthesias (BFP). Unfortunately, five days after admission, the patient suffered a sudden-onset arrhythmia and died before any treatment could be administered.

Although a direct causal relationship cannot be definitively established, there is evidence suggesting that the vaccine may trigger an immune response leading to GBS. Potential mechanisms include molecular mimicry, the production of anti-ganglioside antibodies, and complement activation, as well as adenoviral vector components [78]. From the perspective of molecular mimicry, the antibodies generated in response to the S protein of the vaccine may cross-react with gangliosides on neurons, causing neuronal demyelination. The interaction between the COVID-19 spike protein and gangliosides on cell surfaces raises the possibility of molecular mimicry, where the immune system may cross-react between the viral epitopes and peripheral nerve glycolipids, potentially triggering GBS [166]. Additionally, the mechanism of VITT occurrence after vaccination with ChAdOx and Ad26.COV2.S has been clarified [78]. This insight leads to the proposal that cross-reactivity between adenoviral vector components and peripheral nerve (glyco)proteins might also contribute to GBS [167]. However, the electrostatic interactions that initiate VITT through platelet factor 4 are not easily replicated by peripheral nerve components. Therefore, further research is needed to investigate the cross-reactivity of post-immunization antibodies to different vectors and the potential interactions between adenoviral components and surface molecules of peripheral nerve structures.

Prompt recognition and management are crucial, given the potential for severe neurological complications, including respiratory failure. Although the causal relationship between COVID-19 vaccines and GBS remains uncertain, healthcare providers should maintain vigilance and promptly investigate any suspected cases to ensure timely intervention and patient safety.

### 2.5. Autoimmune Hepatitis (AIH)

Autoimmune hepatitis is a chronic, progressive inflammatory liver disorder driven by autoimmune responses, where the immune system mistakenly attacks the liver cells, leading to inflammation and potential liver damage [168]. Clinical manifestations comprise variable elevations in serum transaminases, hypergammaglobulinemia, positive autoantibodies, and histological findings characterized predominantly by interface hepatitis with infiltration of lymphocytes and plasma cells [169]. Severe cases can quickly advance to cirrhosis and liver failure.

Table S5 provides an overview of diverse case reports detailing the occurrence of AIH post-COVID-19 vaccination [170,171,172,173,174,175,176,177,178,179,180,181,182,183,184,185,186,187,188,189,190,191,192,193,194,195,196,197,198,199]. The implicated vaccines include mRNA, viral vector, and inactivated types, with mRNA vaccines being the most commonly cited and inactivated vaccines the least frequently mentioned. Clayton-Chubb et al. [170] reported the first case of AIH in a 36-year-old male physician following ChAdOx1 nCoV-19 vaccination. Despite being asymptomatic, the patient presented with markedly elevated liver enzymes and positive autoimmune markers weeks after vaccination. Liver biopsy confirmed AIH, prompting treatment with prednisolone and subsequent clinical improvement. Mekritthikrai et al. [171] documented the first case of AIH onset after inactivated COVID-19 vaccination in a previously healthy 52-year-old woman. She developed progressive jaundice and fatigue a week after receiving two doses of the CoronaVac vaccine. Laboratory tests revealed elevated liver enzymes, positive autoimmune markers, and liver cirrhosis on imaging. Treatment with steroids and azathioprine led to resolution of symptoms and normalization of liver function tests within a month. Bril et al. firstly [172] reported a 35-year-old female who developed AIH shortly after receiving the Pfizer-BioNTech mRNA vaccine. She presented with generalized pruritus, choluria, and jaundice 13 days post-vaccination. Despite normal immunoglobulin G levels, liver biopsy findings indicated features consistent with either drug-induced liver injury or autoimmune hepatitis. Treatment with prednisone resulted in clinical improvement.

Most reported cases of vaccine-induced AIH respond well to corticosteroid therapy, resulting in favorable outcomes (see Table S5). However, a 62-year-old diabetic male, who received the ChAdOx1-S vaccine 16 days earlier, presented with a 3-day history of fever, anorexia, and jaundice. He had previously experienced two episodes of jaundice treated with native medication. Despite receiving steroids and undergoing therapeutic plasma exchange, his condition deteriorated, leading to cholestasis and severe coagulopathy. Unfortunately, due to socio-economic constraints, liver transplantation was not pursued, and the patient succumbed to the illness three weeks after admission, despite receiving supportive care [173].

Despite increasing reports linking COVID-19 vaccine administration with AIH, the precise mechanisms remain elusive. The cases outlined suggest that different vaccine types may vary in their potential to induce autoimmunity, possibly through distinct immune modulation mechanisms. mRNA vaccines, utilizing ionizable lipid nanoparticles (iLNPs) that interact with pattern recognition receptors like Toll-like receptors, could initiate immune responses leading to immune-mediated hepatitis. This process may involve innate immune activation triggering inflammatory pathways, potentially resulting in hepatocellular injury and AIH. Viral vector vaccines, on the other hand, may engage adjuvants or interact with pattern recognition receptors such as TLR9, influencing immune responses. Inactivated vaccines might induce immune effects through adjuvants and molecular mimicry phenomena [200,201]. Overall, these findings suggest that COVID-19 vaccination may induce AIH in susceptible populations. Further studies are necessary to elucidate the underlying mechanisms and identify the risk factors associated with AIH post-vaccination.

### 2.6. Type 1 Diabetes Mellitus (T1DM)

Type 1 diabetes mellitus (T1DM) is a chronic autoimmune disorder that affects millions of individuals worldwide, primarily characterized by the autoimmune destruction of pancreatic β cells, which produce insulin—a hormone essential for blood glucose regulation. Patients with T1DM often present with classic symptoms such as polyuria, polydipsia, and weight loss, reflecting the body’s inability to manage glucose levels due to insulin deficiency. This condition requires lifelong management, including insulin therapy, continuous glucose monitoring, dietary adjustments, and regular medical follow-up to prevent acute complications like diabetic ketoacidosis (DKA) and to mitigate the risk of long-term complications [202].

Table S6 provides an overview of some cases where new-onset diabetes was observed following COVID-19 vaccination [203,204,205,206,207,208,209,210,211,212,213,214,215,216]. 50% of the patients were female, and symptoms emerged within days to several weeks post-vaccination, with mRNA vaccines being the most frequently implicated. Key laboratory findings included elevated HbA1c, glucose levels, and the presence of autoantibodies such as anti-GAD, indicating autoimmune conditions. Diagnoses ranged from T1DM to fulminant T1DM (FT1DM). Protocols were diverse, encompassing insulin therapy, corticosteroids, metformin, glimepiride, gemigliptin, and medical nutrition therapy, tailored to manage hyperglycemia and prevent complications. Despite the majority of patients showing improvement, outcomes were not uniformly positive, with one fatality and another case of uncontrolled diabetes, highlighting the heterogeneity of responses and the need for individualized care and close monitoring in the post-vaccination period.

A 36-year-old female without a prior history of diabetes developed symptoms of diabetic ketoacidosis just days after receiving the first dose of a COVID-19 RNA-based vaccine. She was diagnosed with T1D, highlighting the possibility that the vaccine may trigger T1D even in individuals without previous diabetes histories [203]. Reports have emerged detailing rare cases of FT1DM in individuals following COVID-19 vaccination. A 50-year-old man developed FT1DM shortly after receiving the first dose of the COVID-19 inactivated vaccine (CoronaVac) [204]. He presented with polydipsia and polyuria following a fever post-vaccination, and was diagnosed with diabetic ketoacidosis (DKA), managed with insulin infusion. Subsequent tests showed elevated pancreatic enzymes, undetectable C-peptide levels, and negative islet autoantibodies. His HLA alleles associated with FT1DM were positive (DQB1*02:03/03:03 and DRB1*09:01/09:01) [217], confirming the diagnosis. This case indicates that vaccination might trigger autoimmunity in genetically susceptible individuals, leading to irreversible islet cell damage and FT1DM. In a study from Japan [205], a 43-year-old man with a history of stage IIIC malignant melanoma was undergoing nivolumab treatment for 12 months without any previous issues with glucose levels. He received his first SARS-CoV-2 vaccination 35 days before admission with no adverse reactions. Two days after the second vaccination, he developed severe hyperglycemia symptoms. By the twelfth day post-second vaccination, his blood glucose level spiked to 655 mg/dL, and HbA1c increased to 8.0%, with elevated ketone bodies indicating ketosis. Laboratory tests confirmed severely impaired insulin secretion and negative islet-specific autoantibodies, leading to a diagnosis of fulminant T1DM associated with immune checkpoint inhibitor (ICI) treatment. In a separate case reported by Tanaka et al. [206], a 60-year-old man with advanced gastric cancer, who was receiving multiple chemotherapy and immunotherapy regimens including nivolumab and steroid therapy for hypopituitarism-induced hypoadrenocorticism, developed symptoms suggesting adrenal insufficiency after receiving a COVID-19 vaccine. An autopsy revealed loss of islets of Langerhans in the pancreas, confirming fulminant T1DM. Histopathological analysis showed lymphocytic infiltration in the pituitary gland and pancreas, indicating immune-mediated destruction of pancreatic beta cells. Both cases highlight the rare but severe complication of fulminant T1DM following COVID-19 vaccination in patients at risk due to Immunotherapy. The rapid onset of symptoms shortly after vaccination in these patients suggests a possible triggering role of the vaccine in the development of fulminant T1DM.

The mechanism behind these vaccine-induced T1DM cases remains under investigation. MDA5, an innate pathogen recognition protein, has been implicated in the immune response to COVID-19 vaccines. Sakurai et al. suggest that MDA5 can recognize vaccine RNA, triggering type I interferon synthesis, which might disrupt insulin production and pancreatic β-cell function [203]. These cases underscore the need for vigilance among clinicians, particularly for patients on immunotherapy, and suggest that T1DM should be considered a potential adverse effect of COVID-19 vaccination, even in previously healthy individuals. The exact mechanisms linking the vaccine to T1DM onset require further exploration, but the temporal association between vaccination and symptom onset in these cases is compelling.

### 2.7. Myasthenia Gravis (MG)

Myasthenia Gravis (MG) is a relatively uncommon autoimmune disorder where the body mistakenly produces antibodies targeting the nicotinic acetylcholine (ACh) receptors on the postsynaptic side of the neuromuscular junction (NMJ) [218]. This autoimmune attack results in fewer functional ACh receptors, disrupting the normal communication between nerves and skeletal muscles. As a consequence, individuals with MG suffer from muscle weakness and quick exhaustion, especially in muscles responsible for eye movement, facial expression, swallowing, and respiration. With the progression of neurological research, there is an increasing awareness and exploration of these conditions. However, the neurological impacts associated with COVID-19 vaccinations continue to be the subject of ongoing investigation [219].

A review of documented case reports reveals instances of new onset or exacerbation of MG post-COVID-19 vaccination (Table S7) [220,221,222,223,224,225,226,227,228,229,230,231,232,233,234,235,236,237,238,239]. The patients, whose ages span from 13 to 90 years, are predominantly male. They experienced MG symptoms at varying intervals after vaccination, usually within days to several weeks post-vaccination. Most patients received mRNA vaccines (71%, 22 out of 31), suggesting a higher incidence of MG cases linked to mRNA vaccination. Diagnostic findings frequently included elevated levels of antibodies against acetylcholine receptors and EMG results indicative of MG. Treatment approaches were diverse but typically involved medications such as pyridostigmine, prednisone, and immunoglobulins.

The rapid onset of MG is illustrated in a case report involving a 53-year-old man who experienced new-onset MG shortly after receiving his first dose of the ChAdOx1 nCOV-19 (AstraZeneca) vaccine [220]. The patient presented with sudden diplopia, abduction paresis in the right eye, and ptosis in the left eye, which progressed to lateral gaze palsy and ptosis in both eyes. Despite initial treatment with dexamethasone and vitamin B supplements, his symptoms worsened when the steroid was tapered. Further evaluation at a hospital revealed no brain abnormalities but did show elevated anti-AChR antibodies and a decremental response in the repetitive stimulation test, confirming the diagnosis of MG. The patient was treated with pyridostigmine and prednisolone, leading to significant improvement after one month. The report highlights the potential for COVID-19 vaccination to trigger MG and emphasizes the need for further studies to understand the immunopathogenesis of such reactions. Further, in a study involving 27 patients who experienced new-onset immune-mediated disease (IMD) following mRNA/DNA SARS-CoV-2 vaccination, two patients developed new-onset MG after receiving the second dose of the BNT162b2 vaccine. One patient had a moderate case and responded well to treatment, which included plasma exchange and Prednisone, while the other experienced a severe case requiring intubation due to respiratory symptoms [221]. However, not all cases responded positively to treatment. For instance, a 65-year-old male with a history of multiphasic acute disseminated encephalomyelitis (ADEM) presented with new neurological symptoms three days after receiving the third dose of the Pfizer-BioNTech COVID-19 vaccine. Despite initial treatment with high-dose corticosteroids and intravenous immunoglobulin (IVIG), his condition worsened, leading to the development of ocular myasthenia gravis and subacute thyroiditis. Plasmapheresis was subsequently administered, resulting in clinical improvement and resolution of symptoms [222].

While these reports suggest that COVID-19 vaccination may trigger MG, the underlying immune mechanisms remain inadequately understood. Given the unclear pathophysiological connections between MG and the vaccine, additional research is imperative.

### 2.8. Alopecia Areata (AA)

Alopecia areata (AA) is an autoimmune disorder characterized by patchy, nonscarring hair loss, affecting approximately 2% of the population at some point in their lives [240,241]. It accounts for 18.2% of hair loss cases in primary care settings and is caused by an autoimmune attack on hair follicles, disrupting the anagen growth phase [242]. The condition can escalate to alopecia totalis and alopecia universalis, involving complete scalp and body hair loss, respectively. The etiology of AA is uncertain, but it is widely believed to involve immune system dysfunction and genetic predisposition.

Table S8 summarizes recent case reports of AA following COVID-19 vaccination [243,244,245,246,247,248,249,250,251,252,253,254,255,256,257,258,259,260,261,262]. The findings reveal a higher prevalence of AA in females and suggest that AA can occur across a broader age spectrum. Lee et al. [243] reported on an 80-year-old man without prior history of alopecia or autoimmune disorders who developed symptoms shortly after receiving the first dose of the BNT162b2 mRNA vaccine. Symptoms began 7 days after the initial dose and worsened following the second dose, resulting in rapid facial hair loss and later widespread scalp involvement diagnosed as AA. Despite treatment with topical clobetasol foam, the condition progressed to alopecia areata totalis. Trichoscopy revealed characteristic features such as exclamation point hairs and broken hairs. Martora et al. [244] discussed a 7-year-old girl who developed AA and herpes zoster 20 days after the second dose of the BNT162b2 mRNA vaccine. Adverse reactions to the vaccine can manifest shortly after vaccination or as delayed cutaneous reactions. This case highlights the simultaneous onset of alopecia areata and herpes zoster, suggesting potential mechanisms such as vaccine-induced immunomodulation triggering herpes zoster and autoimmune responses leading to alopecia areata.

Table S8 also shows an association between prior AA or thyroid dysfunction and the risk of AA development following COVID-19 vaccination. This underscores the critical need to consider pre-existing autoimmune conditions when assessing the potential risks in susceptible populations. The mRNA vaccine, widely used in reported AA cases, may involve allergenic components like polyethylene glycol (PEG) [263]. Moreover, heterologous prime-boost COVID-19 vaccines have been implicated in triggering multiple autoimmune reactions in some individuals. For instance, a 27-year-old woman developed SLE and AA after receiving heterologous prime-boost COVID-19 vaccines [245]. Initially, she presented with bullous, exulcerated skin lesions and systemic symptoms post-ChAdOX1 nCoV-19/AZD1222 vaccination, followed by alopecia after BNT162b2 vaccine administration. Laboratory findings confirmed SLE diagnosis, and trichoscopy indicated AA with characteristic features such as yellow and black dots, dystrophic hair, and white hairs of replication. These observations strongly suggest that COVID-19 vaccination may induce complex autoimmune responses in susceptible individuals. Additionally, some patients experienced exacerbated AA following their second vaccine dose. Abdalla et al. [246] reported a case of a 63-year-old woman with AA who experienced initial patchy hair loss within two weeks of the first vaccine dose, progressing to complete hair loss six weeks after the second dose. This case underscores the importance for dermatologists to carefully evaluate the necessity of subsequent vaccine doses in patients presenting AA symptoms after initial vaccination.

The exact mechanisms by which COVID-19 vaccinations induce AA are still unclear, but potential factors include cross-reactivity between antigens, adjuvant effects, and the activation of proinflammatory cascades that disrupt hair follicle growth and immune privilege [264,265]. Further research is essential to elucidate the pathogenesis of vaccine-associated AA and to develop more effective preventive and therapeutic strategies.

### 2.9. Antiphospholipid Syndrome (APS)

Antiphospholipid syndrome (APS) is a unique autoimmune disorder characterized by the presence of antiphospholipid antibodies (aPL), which significantly increase the risk of recurrent thrombosis and pregnancy-related complications [266]. This acquired condition is marked by persistent aPL, including lupus anticoagulant, anticardiolipin antibodies, and anti-β2-glycoprotein I antibodies, contributing to its clinical manifestations such as arterial, venous, or small vessel thrombosis, and obstetric issues including recurrent pregnancy loss, preeclampsia, and preterm birth [267]. Diagnosing APS necessitates the detection of aPL on at least two separate occasions spaced by 12 weeks or more, accompanied by clinical thrombotic or obstetric events. Notably, lupus anticoagulant is a particularly significant predictor of APS-related complications [268].

Table S9 summarizes several case reports [29,269,270,271,272] of individuals who developed symptoms suggestive of APS or related conditions post-COVID-19 vaccination. The cases involve both mRNA and inactivated vaccines, with symptom onset ranging from 1 day to 6 weeks after vaccination. Molina-Rios et al. reported a 42-year-old female with a history of three pregnancies, two of which resulted in spontaneous abortion. Two weeks after receiving the first dose of mRNA vaccine, she developed inflammatory polyarthralgia, bilateral synovitis, and bilateral Achilles tendonitis. Given the laboratory data and imaging findings, SLE and secondary APS were suspected [29].

In addition, there are two reported cases of catastrophic antiphospholipid syndrome (CAPS) reported. Moreno-Torres et al. [269] reported a case of CAPS following mRNA COVID-19 vaccination. The patient, a 27-year-old female with a history of selective immunoglobulin A deficiency and mild COVID-19 symptoms, developed fever, digital ischemia, and abdominal pain 36 h after receiving the first dose of BNT162b2 mRNA vaccine. Laboratory tests revealed the presence of lupus anticoagulant. The patient received treatment with low-dose prednisone, hydroxychloroquine, adjusted low-molecular-weight heparin (LMWH), and thrice-weekly hemodialysis. Jinno et al. [270] also reported a case of CAPS in a 71-year-old female patient who developed multiple organ thrombosis shortly after receiving the first dose of the BNT162b COVID-19 vaccine by BioNTech/Pfizer. The patient exhibited triple-positive aPLs and had a history of coronary artery disease. Despite initial treatment for acute myocardial infarction, the patient continued to experience thromboembolic events, leading to a diagnosis of CAPS. The patient was treated with methylprednisolone, plasmapheresis, and long-term anticoagulation, showing improvement in symptoms. The authors propose that the vaccine’s spike protein subunits might induce the generation of aPLs, potentially exacerbated by the patient’s essential thrombocythemia.

Overall, while the benefits of COVID-19 vaccination generally outweigh the risks, ongoing research is essential to fully understand the long-term implications of vaccination on APS. However, it is important to note that these cases represent a small number of individuals and do not necessarily indicate a causal relationship between COVID-19 vaccination and the development of APS.

### 2.10. ANCA-Associated Vasculitis (AAV)

ANCA-associated vasculitis (AAV) is a rare autoimmune condition that primarily affects small blood vessels, causing inflammation and damage to vessel walls that can result in ischemia and organ damage if not promptly addressed. The disease is linked to antineutrophil cytoplasmic antibodies (ANCAs) that target neutrophil granule proteins, such as proteinase 3 (PR3-ANCA) and myeloperoxidase (MPO-ANCA), triggering neutrophil activation and subsequent vessel inflammation. AAV encompasses GPA (granulomatosis with polyangiitis), MPA (microscopic polyangiitis), and EGPA (eosinophilic granulomatosis with polyangiitis), each with distinct clinical features and ANCA patterns [273].

Here, we summarize 53 cases of AAV that occurred following COVID-19 vaccination (Table S10) [25,274,275,276,277,278,279,280,281,282,283,284,285,286,287,288,289,290,291,292,293,294,295,296,297,298,299,300,301,302,303,304,305]. Among the 53 cases of AAV post-COVID-19 vaccination, 38 involved the use of mRNA vaccines, 12 involved viral vector vaccines, and 3 involved inactivated vaccines. The majority of patients were treated with corticosteroids, and a subset also received additional interventions, including hemodialysis (3.8%), plasma exchange (18.8%), cyclophosphamide (45.3%), and Rituximab (49%). Suzuki et al. [274] described a 72-year-old man who developed severe myeloperoxidase-antineutrophil cytoplasmic antibody (MPO-ANCA)-associated vasculitis with kidney involvement after Pfizer-BioNTech COVID-19 vaccination. He presented with acute kidney injury, fever, fatigue, and appetite loss following his second vaccine dose. Diagnostic tests revealed elevated MPO-ANCA levels consistent with vasculitis. Treatment included high-dose steroids, rituximab, and hemodialysis, leading to clinical improvement. This case underscores the importance of considering vasculitis in patients with persistent systemic symptoms or renal abnormalities post-vaccination, although further research is needed to establish causation. The onset of AAV could also occur after a prolonged duration. Dourado et al. [275] detailed the case of a 47-year-old man who developed increasing fatigue, anorexia, and abdominal pain three months after receiving the second dose of Pfizer-BioNTech COVID-19 vaccine. Despite initial treatment for suspected prostatitis, his renal function deteriorated, prompting hospitalization. Subsequent investigations revealed pauci-immune necrotizing crescentic glomerulonephritis with positive ANCA-MPO antibodies. Treatment included plasmapheresis, cyclophosphamide, and steroids, initially stabilizing him but ultimately requiring maintenance hemodialysis and prednisolone therapy.

The hypothesis that vaccines, particularly COVID-19 vaccines, may induce vasculitis or autoimmune phenomena is being explored, drawing parallels with the “Autoimmune/Inflammatory Syndrome Induced by Adjuvants” (ASIA) framework [306]. ASIA encompasses a range of conditions linked to exposure to immune adjuvants, such as siliconosis, Gulf War syndrome, macrophagic myofasciitis syndrome, and post-vaccination phenomena. Unlike the relatively immediate onset of symptoms observed in ASIA, systemic vasculitis following COVID-19 vaccination often exhibits a longer latency period, with symptoms manifesting weeks, months, or even years after vaccination. The mechanisms through which COVID-19 vaccines might trigger AAV are not yet fully understood. However, the role of adjuvants, such as aluminum salts commonly used in vaccines, in potentially triggering autoimmune responses is well-documented. Adjuvants enhance the immune response to antigens by stimulating both the innate and adaptive immune systems, which can lead to an overactive or misdirected immune reaction. This is supported by findings in ASIA, where adjuvants have been shown to induce autoimmune diseases in animal models and humans.

Further research is essential to elucidate the specific pathways and immune mechanisms involved in vaccine-induced AAV. This includes understanding the role of adjuvants in the development of autoantibodies and the subsequent inflammatory responses. The research on ASIA provides a valuable framework for exploring these questions, highlighting the importance of genetic predisposition, the timing and duration of exposure to adjuvants, and the potential for co-exposure to multiple triggers.

## 3. Mechanisms of Autoimmune Diseases Induced by COVID-19 Vaccines

**Figure 2:**
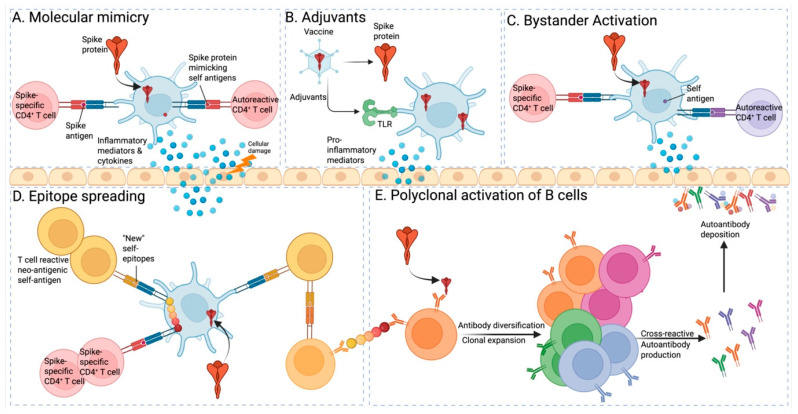
(**A**) Molecular mimicry. Autoreactive T cells can be activated through cross-reactive recognition when a viral antigen shares structural or sequence similarities with a self-antigen. This phenomenon, known as molecular mimicry, occurs when the immune system mistakes viral proteins for self-proteins due to their resemblance. This activation can lead to an immune response against both the virus and the host’s own tissues, potentially triggering or exacerbating autoimmune diseases. (**B**) Adjuvants. Adjuvants in vaccines, in the context of SARS-CoV-2 vaccines, activate APCs through Toll-like receptors (TLRs). This activation can inadvertently trigger autoimmune responses if similarities between vaccine components and self-antigens lead to the activation of autoreactive T cells. (**C**) Bystander activation. Spike-specific T cells can contribute to bystander activation. This occurs when self-antigens, released from damaged tissue, are taken up by activated antigen-presenting cells (APCs), processed, and presented alongside viral antigens to autoreactive T cells. This phenomenon, known as bystander activation, leads to the stimulation of pre-existing autoreactive T cells, potentially initiating autoimmune diseases. Additionally, infections can activate a subset of T cells through microbial superantigens, some of which may recognize self-antigens. Viral infections are particularly effective at activating APCs, such as dendritic cells, further exacerbating immune responses. (**D**) Epitope spreading. Autoreactive T cells, initially activated by viral antigens that mimic self-antigens presented by APCs, can expand their response to attack other self-antigens—a phenomenon known as epitope spreading. Superantigens directly bind to T-cell receptors and MHC class II molecules on APCs, inducing a broad activation of T cells. This indiscriminate activation may result in exaggerated immune responses, cytokine release, and potential immunopathological consequences. (**E**) Polyclonal activation of B cells. Following vaccination, polyclonal activation of B cells triggers a cascade of events. Initially, B cells recognize viral antigens, leading to their proliferation and differentiation into antibody-producing plasma cells. Some antibodies generated may cross-react with self-antigens, resulting in the production of autoantibodies. These autoantibodies can potentially deposit in tissues, contributing to autoimmune reactions.

COVID-19 vaccines work by activating the immune system through the introduction of viral protein segments, such as the spike protein, or the virus’s genetic material, like mRNA. While these vaccines have proven effective in fighting the virus, there is growing evidence suggesting a possible link between COVID-19 vaccination and autoimmune disorders (Table 2). To understand this potential connection, several mechanisms have been proposed, such as molecular mimicry, adjuvants, bystander activation, epitope spreading, and polyclonal B cell activation (Figure 2). Although these mechanisms remain hypothetical, they provide a framework for understanding how vaccines might rarely break immune tolerance in genetically susceptible individuals.

Recent advances in systems vaccinology have begun to clarify the molecular underpinnings of these processes. Innate recognition of RNA or vector components via TLR7/9 and RIG-I–like receptors induces robust type I IFN and IL-6 responses that enhance antigen presentation and costimulatory signaling [307,308]. In genetically susceptible individuals, these cytokine pathways can amplify autoreactive Tfh and B-cell activation, leading to breakage of peripheral tolerance [309,310]. Moreover, adjuvants such as alum or Matrix-M activate the NLRP3 inflammasome and NF-κB, driving polyclonal B-cell responses and further immune amplification [311,312]. Collectively, these mechanisms provide a biological context for understanding the rare autoimmune manifestations temporally associated with vaccination, integrating innate sensor engagement, cytokine cascades, and adaptive immune dysregulation.

### 3.1. Molecular Mimicry

Molecular mimicry, where foreign pathogens mirror the host’s cells, can lead to a misguided immune response attacking the host’s tissues. This is considered a key mechanism contributing to autoimmunity development [313]. Wucherpfennig and Strominger’s study [313] initially focused on T-cell-mediated autoimmunity, particularly in the context of molecular mimicry. They discovered that certain viral peptides can activate T cell clones specific to myelin basic protein (MBP), potentially instigating an autoimmune response. Their study suggests that activation of these T cells could lead to autoimmune diseases like multiple sclerosis and indicates that a group of common viral pathogens could trigger the autoimmune process rather than a single virus. This process is influenced by host genetics, exposure to microbiota, and environmental chemicals. Traditionally, it has been associated with T-cell-mediated conditions, but recent research indicates the role of B-cell responses and the production of autoantibodies [314]. Furthermore, instances of vaccine-induced autoimmune diseases linked to molecular mimicry and immune cross-reactivity include the correlation of the influenza A virus (H1N1) vaccine with Guillain–Barré syndrome, the hepatitis B virus vaccine with multiple sclerosis, and the human papillomavirus (HPV) vaccine with systemic lupus erythematosus. Similar mechanisms might play a role in autoimmune diseases following SARS-CoV-2 vaccination [10].

Khavinson et al. [12] investigated the structural similarities between SARS-CoV-2 proteins and human proteins to understand the potential mimicry and autoimmune responses during SARS-CoV-2 infection. They found over two dozen hepta- and octamers in the SARS-CoV-2 spike protein that were homologous to human proteins. These sequences, projecting from the virus particle, could potentially mislead the immune system. Conversely, the envelope protein’s homologous sequences were all within the viral transmembrane domain, suggesting they could provoke an autoimmune response after the virus’s destruction. The study also investigated nonstructural proteins and found that some, such as ORF3a, ORF7a, ORF7b, ORF8, and ORF9b, may contribute to an autoimmune response. Specifically, ORF7b might be involved in olfactory dysfunction, and the S protein might be involved in taste perception dysfunction. Therefore, it appears plausible that certain SARS-CoV-2 proteins may engage in molecular mimicry, potentially delaying the immune response and triggering autoimmune processes.

Another study [312] found a significant and unexpected overlap of heptapeptide sequences between the SARS-CoV-2 spike glycoprotein and human proteins. This overlap was not observed in species that do not exhibit significant pathology following SARS-CoV-2 infection, like domestic animals, rabbits, and some primates. These findings support molecular mimicry as a potential mechanism contributing to diseases associated with SARS-CoV-2. The authors suggest that this has implications for vaccine design and testing, emphasizing the need for careful choice of laboratory animals in preclinical studies to accurately reflect potential cross-reactivity and subsequent autoimmunity. They propose that older mice might be the best animal model for testing an anti-SARS-CoV-2 spike glycoprotein vaccine for human use. They endorse the development of COVID-19 vaccines based on minimal immune determinants unique to pathogens and absent in the human proteome for safety and efficacy.

### 3.2. Adjuvants

Adjuvants are compounds added to vaccines to enhance the strength, duration, and specificity of the immune response to antigens. They amplify the antigen-specific immune response without possessing inherent antigenicity, thereby boosting the overall effectiveness of the vaccine [315]. Toll-like receptor ligand adjuvants (TLRLs) serve as immunostimulants integrated into vaccines to initiate innate immune responses [316,317]. Interestingly, studies suggest that robust antibody responses can still be elicited even in the absence of Toll-like receptor signals [318]. The autoimmune/inflammatory syndrome induced by adjuvants (ASIA), introduced by Shoenfeld and Agmon-Levin in 2011, encompasses a spectrum of autoimmune conditions triggered by exposure to various adjuvants [319]. Substances such as silicon and heavy metals like mineral oil, guaiacol, iodine gadital, mercury, and titanium have been implicated in ASIA, although they are not typically used in vaccines [320]. mRNA-based lipid nanoparticle (LNP) vaccines represent an innovative platform adopted by leading COVID-19 vaccines. LNPs contain a blend of phospholipids, cholesterol, PEGylated lipids, and cationic or ionizable lipids. They exhibit potent adjuvant properties while safeguarding mRNA from degradation, facilitating intracellular delivery, and promoting escape from endosomes [321]. Notably, mRNA vaccines serve dual roles as both antigens and adjuvants, activating intracellular Toll-like receptors and membrane-bound inflammatory components to induce inflammation and immune responses [322]. The NLRP3 inflammasome plays a critical role in innate and adaptive immunity and is associated with various autoimmune conditions such as rheumatoid arthritis, SLE, Sjögren’s syndrome, ankylosing spondylitis, and axial spondyloarthritis [323]. However, further research is necessary to validate the potential role of adjuvants in triggering autoimmune diseases following COVID-19 vaccination. Understanding the implications of adjuvants on vaccine-induced autoimmune disorders is crucial for enhancing vaccine safety and optimizing the management of autoimmune conditions.

### 3.3. Bystander Activation

Bystander activation, a hallmark of antigen-nonspecific immunity, occurs when infection stimulates innate immune responses or leads to the release of self-antigens [324]. As a result, self-reactive T cells are activated, leading to cytokine secretion, inflammation, and heightened production of self-reactive lymphocytes. Research by Tough et al. [324] demonstrated that during viral infections, memory T cells can proliferate significantly without substantial engagement of their T cell receptors (TCRs). This cytokine-driven proliferation, observed in CD44hi CD8+ T cell subsets, highlighted the critical role of type I interferon (IFN-I), such as induced by poly(I), in stimulating non-antigen-specific responses. Intermittent exposure to IFN-I during viral infections is sufficient to promote durable memory conveyed by CD8^+^ cells [325]. Boyman et al. additionally indicate that IL-2 and other cytokines may contribute to bystander activation of CD4+ T cells, extending our understanding beyond the well-established phenomenon in CD8+ T cells [326]. In vaccines containing adjuvants or when adjuvants are used independently, T cells can be activated through bystander pathways. However, the migration of these activated self-reactive cells to inflammatory sites and their subsequent cytokine secretion may potentially trigger autoimmune diseases [327], such as COVID-19 RA, SLE, and T1DM. Therefore, bystander activation likely plays a role in the autoimmune phenomena observed after COVID-19 vaccination. Further research is essential to clarify the impact of bystander activation on vaccine-induced autoimmune diseases and to develop strategies to mitigate potential adverse effects.

### 3.4. Epitope Spreading (ES)

Epitope spreading (ES) involves the development of immune responses to epitopes that are distinct from and do not cross-react with the dominant epitope [328]. This phenomenon can occur either within the same molecule (targeting other epitopes on the same protein) or between different molecules (targeting epitopes on different proteins). Cornaby et al. [329] illustrate that both intra- and inter-molecular B cell epitope spreading significantly contribute to the pathogenesis of several autoimmune diseases, such as systemic lupus erythematosus (SLE), rheumatoid arthritis (RA), Graves’ disease, and multiple sclerosis (MS). This process highlights how the autoimmune response diversifies over time, extending to additional self-antigens beyond the initial target. Such diversification involves both intra- and intermolecular spreading of autoantibodies, which exacerbates disease severity. Epitope spread, also known as determinant cascade, refers to the amplification of an immune response to secondary epitopes that were not originally targeted by therapy [330]. This dynamic process may persist and broaden over time. In the context of vaccines, antigen spread can result from the immune response to the vaccine antigen, potentially activating autoreactive T cells or B cells that recognize self-antigens, thereby contributing to the onset of autoimmune diseases. Further research is crucial to understand the role of epitope spreading in COVID-19-vaccine-induced autoimmune diseases and to develop strategies for mitigating potential adverse effects.

### 3.5. Polyclonal Activation of B Cells

Polyclonal activation refers to a nonspecific immune response characterized by the stimulation of a wide range of B cells [331]. Polyclonal activators stimulate a significant number of B or T lymphocytes, promoting their proliferation and inducing functions typically triggered by antigen-mediated activation. These activators include various agents such as plant-derived lectins (e.g., PHA, ConA, PWM), bacterial LPS acting via Toll-like receptor 4, and superantigens like staphylococcal enterotoxin B (SEB), which broadly activate T cells. For example, this reaction often occurs when a high concentration of antigen binds to the LPS receptor on B cells, triggering the production of low-affinity IgM-like antibodies, with LPS notably serving as a polyclonal activator in this context [332]. Additionally, adjuvants and superantigens can induce polyclonal activation, potentially activating autoreactive immune cells and promoting autoimmune responses, which may lead to autoimmune diseases [333]. For example, Fuchs’ complete adjuvant, often combined with tissue homogenates or purified autoantigens, is used to induce experimental autoimmune diseases. In vaccine formulations, adjuvants are included to enhance immunogenicity and promote sustained immune responses to non-replicating, inactivated, or subunit antigens [334]. This process can lead to excessive production of autoantibodies and cytokines, contributing to inflammation and tissue damage. In autoimmune diseases like systemic lupus erythematosus (SLE) or rheumatoid arthritis, polyclonal activation plays a critical role in perpetuating immune responses against self-antigens, resulting in chronic inflammation and tissue destruction. This underscores the complexity of autoimmune diseases, such as systemic lupus erythematosus, rheumatoid arthritis, and Sjögren’s syndrome, necessitating further research to understand the mechanisms underlying polyclonal activation and its specific contributions to disease pathogenesis [335]. Concerns have also been raised in the context of COVID-19 vaccines regarding potential polyclonal activation and subsequent development of autoimmune diseases.

The aforementioned analysis explores the possible connection between COVID-19 vaccinations and the onset of autoimmune disorders. According to the molecular mimicry hypothesis, the resemblance between the spike protein in COVID-19 vaccines and specific human tissue proteins might contribute to the exacerbation or initiation of autoimmune conditions. Moreover, the inflammatory responses elicited by COVID-19 vaccines could disrupt immune system balance, particularly with mRNA vaccines, which are known to trigger proinflammatory cytokines like type I interferons, potentially leading to a breakdown in immune tolerance [336,337]. Integration of clinical and mechanistic observations suggests that Guillain–Barré syndrome likely reflects molecular mimicry and bystander activation, whereas lupus-like syndromes correspond to type I IFN–driven polyclonal B-cell activation and epitope spreading. Similarly, autoimmune hepatitis may involve combined effects of adjuvant-mediated inflammasome activation and antigenic mimicry between viral and hepatic proteins. These cross-sectional insights strengthen the translational interpretation of vaccine-associated immune phenomena. Nonetheless, these proposed mechanisms remain theoretical and require further investigation to substantiate the relationship between autoimmune diseases and COVID-19 vaccinations and to understand the underlying processes involved.

## 4. Conclusions

Vaccination has been one of the greatest achievements in modern medicine, saving millions of lives annually through the prevention of infectious diseases. From an immunological perspective, new occurrences of autoimmune manifestations have been observed. Initially, we discussed the autoimmune diseases such as systemic lupus erythematosus, rheumatoid arthritis, type 1 diabetes, and autoimmune hepatitis, with molecular mimicry proposed as a possible mechanism for inducing autoimmunity. Current evidence suggests that while COVID-19 vaccination may transiently influence immune regulation, establishing a causal relationship remains challenging. Reports of vaccine-induced autoimmune conditions are primarily limited to case studies and small series, lacking comprehensive documentation and large-scale, population-based cohort studies to substantiate these findings [5,338]. Moreover, many autoimmune diseases exhibit long latency periods between the emergence of autoantibodies and symptom onset, making it difficult to determine whether these events are vaccine-induced or coincidental.

Our study has summarized potential molecular mechanisms underlying the impact of COVID-19 vaccination on the risk of autoimmune diseases, emphasizing the importance of cautious interpretation [339]. Unlike prior reviews, this work integrates clinical patterns with mechanistic immunology, creating a translational framework that connects vaccine components, host immune pathways, and autoimmune susceptibility. Through this approach, we synthesize clinical case patterns with molecular insights—including innate sensor activation (TLR7/9, RIG-I), cytokine signaling (type I interferon and IL-6), and adaptive immune processes such as molecular mimicry, epitope spreading, and polyclonal B-cell activation—to clarify how distinct vaccine platforms may differentially modulate immune tolerance. This integrated perspective provides a clearer mechanistic understanding of the rare autoimmune events temporally associated with COVID-19 vaccination. These mechanisms may be amplified in genetically predisposed or previously primed individuals, resulting in transient immune dysregulation or clinical autoimmunity in a very small subset of the population. Importantly, the frequency of such events remains exceedingly low relative to the billions of vaccine doses administered globally, and their occurrence does not outweigh the profound public health benefits of vaccination.

Autoimmune phenomena observed after COVID-19 vaccination resemble rare events reported following influenza, hepatitis B, and HPV vaccination, where shared mechanisms such as interferon signaling and adjuvant-mediated immune activation have been implicated [11,340]. However, the use of novel mRNA and adenoviral platforms introduces unique immunostimulatory profiles that warrant ongoing evaluation [307]. Comparative analysis of these vaccine systems underscores that while vaccine-triggered autoimmune reactions are exceedingly rare, understanding their shared pathways may inform the rational design of future vaccine platforms with optimized immune safety.

Moving forward, it is imperative to establish global immunovigilance frameworks to identify and monitor potential autoimmune manifestations following vaccination. Large-scale, longitudinal studies integrating genetic, epigenetic (methylomic), transcriptomic, and immune-repertoire analyses will help distinguish causation from coincidence and reveal predictive biomarkers of susceptibility [339]. Particular attention should be given to dissecting the interplay of molecular mimicry, epitope spreading, and polyclonal B-cell activation in individuals with predisposing genetic or environmental backgrounds [341].

Looking ahead, understanding the mechanistic basis of vaccine-associated autoimmunity is essential for strengthening vaccine design and global immunization strategies. Future work should prioritize integrated clinical and mechanistic research, focusing on how vaccine components—such as mRNA molecules, viral vectors, and adjuvants—activate innate sensors (TLR7/9, RIG-I) and downstream cytokine networks (type I IFN, IL-6) in genetically or environmentally predisposed individuals. Mechanistic pathways, including molecular mimicry, epitope spreading, and polyclonal B-cell activation, likely converge to break immune tolerance in this small subset of subjects. Comprehensive multi-omics and immune-repertoire profiling across vaccine platforms (mRNA, adenoviral vector, inactivated, and protein-subunit) will be essential to distinguish causal from coincidental associations and identify biomarkers predictive of susceptibility. Establishing long-term surveillance cohorts and global immunovigilance frameworks can enable early detection of rare autoimmune outcomes while preserving public confidence. Ultimately, these insights will inform the rational design of next-generation vaccines that maintain potent protection with minimal autoimmune risk, ensuring safer and more targeted mass vaccination strategies for future pandemic preparedness.

Ultimately, the collective evidence underscores that the benefits of COVID-19 vaccination far exceed its risks. The lessons learned from these rare autoimmune events not only strengthen our understanding of immune regulation but also provide crucial insight for future pandemic preparedness. Through vigilant monitoring, mechanistic research, and transparent communication, the scientific community can ensure that vaccination continues to remain both a cornerstone of public health and a model for safe, precision immunization.

## Figures and Tables

**Table 1:** Summary of COVID-19 Vaccine–Associated Autoimmune Events by Disease Type.

Autoimmune Disease	Common Vaccine Type(s) Implicated	Median Age (Years)	Sex Ratio (F:M)	Typical Onset (After Vaccination)	Hallmark Laboratory Findings	Common Treatment	Overall Outcome
Systemic Lupus Erythematosus (SLE)	mRNA (majority), Adenoviral vector	22–79	~2:1	2–14 days (median ≈ 7 days)	ANA+, anti-dsDNA+, low C3/C4, ↑ ESR/CRP	Prednisone ± HCQ ± MMF	Most improved; few lupus nephritis cases
Rheumatoid Arthritis (RA)	mRNA, Adenoviral vector, Inactivated	32–88	~3:1	2–20 days	↑ CRP/ESR, RF+, anti-CCP+, ANA+	MTX, HCQ, steroids	Majority improved; rare recurrence
Vaccine-Induced Thrombotic Thrombocytopenia (VITT)	Adenoviral vector (dominant)	25–75	1:1	5–21 days	↓ Platelets, ↑ D-dimer, anti-PF4+	IVIG, anticoagulants, steroids	~25% mortality reported
Guillain–Barré Syndrome (GBS)	Adenoviral vector > mRNA > Inactivated	20–90	~1.5:1	5–28 days	↑ CSF protein (albuminocytologic dissociation)	IVIG ± plasma exchange	Mostly recovered; some partial
Autoimmune Hepatitis (AIH)	mRNA > Adenoviral vector > Inactivated	35–85	~3:1	7–28 days	↑ AST/ALT/bilirubin, ANA+, ASMA+, high IgG	Prednisone ± azathioprine	Most recovered with treatment
Type 1 Diabetes Mellitus (T1DM)	mRNA (predominant), Adenoviral vector, Inactivated	36–73	~1.5:1	3–28 days	Hyperglycemia, ketoacidosis, ↓ C-peptide, anti-GAD+	Insulin ± supportive therapy	Majority improved

**Table 2:** Aggregate Clinical Trends by Mechanistic Association.

Proposed Mechanism	Representative Diseases	Supporting Clinical Patterns	Key Immunological Features
Molecular mimicry	GBS, AIH, RA	Autoantibody overlap (anti-PF4, anti-CCP)	Cross-reactive epitopes between Spike and host proteins
Bystander activation	Thyroiditis, SLE	Occurs after strong innate cytokine response	↑IL-6, IFN-α, TLR7/9 activation
Epitope spreading	SLE, RA	Expansion of antibody repertoire after initial trigger	Secondary autoantibody diversification
Polyclonal B-cell activation	SLE, AIH	Broad ANA/anti-dsDNA/anti-Sm induction	Excessive IL-6 and IFN signatures
Adjuvant effects (ASIA)	AIH, arthritis, myositis	Delayed onset, strong adjuvant formulations	Innate immune overactivation by squalene/alum/Matrix-M

## Data Availability

No new data were created or analyzed in this study.

## Supplementary Materials

The following supporting information can be downloaded at: https://www.mdpi.com/article/10.3390/vaccines13111112/s1, Table S1: Summary of case reports on SLE after COVID-19 vaccination; Table S2: Summary of case reports on RA after COVID-19 vaccination; Table S3: Summary of case reports on VITT after COVID-19 vaccination; Table S4: Summary of case reports on GBS after COVID-19 vaccination; Table S5: Summary of case reports on AIH after COVID-19 vaccination; Table S6: Summary of case reports on T1DM after COVID-19 vaccination; Table S7: Summary of case reports on MG after COVID-19 vaccination; Table S8: Summary of case reports on AA after COVID-19 vaccination; Table S9: Summary of case reports on APS after COVID-19 vaccination; Table S10: Summary of case reports on AAV after COVID-19 vaccination.

## Abbreviations

The following abbreviations are used in this manuscript:

AID	Autoimmune diseases
SLE	Systemic lupus erythematosus
RA	Rheumatoid Arthritis
APS	Antiphospholipid Syndrome
AOSD	Adult-Onset Still’s Disease
AAV	ANCA-Associated Vasculitis
GCA	Giant Cell Arteritis
AIH	Autoimmune Hepatitis
T1DM	Type 1 Diabetes Mellitus
GBS	Guillain–Barré Syndrome
MG	Myasthenia Gravis
AA	Alopecia Areata
ATD	Autoimmune thyroid disease
VITT	Vaccine-induced Immune Thrombotic Thrombocytopenia
RF	Rheumatoid Factor
TLRs	Toll-like receptors
IVIG	Intravenous immune globulin
PEX	Plasma exchange
