# Supplementary material for: Vaccine-Associated Autoimmunity: From Clinical Signals to Immune Pathways
Source: Vaccines (Basel). 2025 Oct 30;13(11):1112. doi: 10.3390/vaccines13111112 (PMC12656999; doi:10.3390/vaccines13111112)
Supplement: Supplementary file 1 [file vaccines-13-01112-s001.zip › vaccines-3921342-supplementary.pdf]

Table S1 Summary of case reports on SLE after COVID-19 vaccination

Author	Age	Sex	Timeline of Symptom Onset	Type of Vaccine	Laboratory Features	Diagnosis	Treatment	Outcome	Ref	Case #
Patil et al.	22	F	10 days after 2nd dose	Adenoviral vector	Positive ANA, elevated anti-dsDNA, thrombocytopenia, high ESR, CRP	New-onset SLE	HCQ, MMF, prednisolone	Improvement of symptoms	[18]	1
Lemoine e et al	68	F	2 days after 1st dose	mRNA	Positive ANA, anti-dsDNA Ab, ESR, CRP, + p-ANCA with anti-MPO specificity	New-onset SLE	Prednisone with taper, azathioprine, methotrexate	Improvement of symptoms	[19]	1
Moriyama et al.	53	M	9 days after 3rd dose	mRNA	Positive ANA, anti-dsDNA, anti-U1-RNP, anti-SS-A, anti-β2 glycoprotein 1 IgG, and lupus anticoagulant	New-onset SLE	Prednisolone, HCQ	Improvement of symptoms	[20]	1
Raviv et al.	24	M	2 days after 1st dose	mRNA	Positive ANA, anti-chromatin, anti-ribosome P protein Ab, and low C3 level	New-onset SLE	HCQ, mometasone furoate cream, etoricoxib	Improvement of symptoms	[21]	1
Kaur et al.	54	M	2 weeks after2nd dose	mRNA	Positive ANA, anti-dsDNA, anti-Ro/SSA, anti-Sm, anti-	New-onset SLE	Prednisone and MMF	Improvement of symptoms	[22]	1

					RNP, low C3, C4					
Baez-Negron et al.	27	F	2 weeks after 2nd dose	mRNA	Positive ANA, anti-dsDNA, anti-Ro, low C4, high ESR	New-onset SLE	HCQ, prednisone, MMF	Favorable response	[23]	1
Kreuter et al.	79	M	10 days after 1st dose	mRNA	Positive ANA, anti-Ro, anti-La	New subacute cutaneous lupus	HCQ, intravenous corticosteroids	Complete resolution	[24]	1
Matsuda et al.	32	F	2-3 days after 3rd dose	mRNA	Positive ANA, antiribonucleoprotein antibody, anti-Sm antibody and anti-SS-A antibody	New-onset SLE	Oral glucocorticoid and HCQ	Improvement of symptoms	[25]	1
N AM et al.	22	F	1 week after 1st dose	mRNA	Positive ANA, anti-dsDNA, high ESR	New-onset SLE	Pulse methylprednisolone with prednisone taper, HCQ, and azathioprine	Improvement of symptoms	[26]	1
Nune et al.	24	M	2 weeks after 2nd dose	mRNA	Positive ANA, anti-dsDNA Ab, elevated CRP, and low C3 and C4 levels	New-onset SLE	Methylprednisolone and cyclophosphamide, followed by oral prednisolone and HCQ	Improvement of symptoms	[27]	1

Sagy et al	24	M	1 week after 1st dose	mRNA	Positive ANA, Ribosomal P, chromatin, low C3	New-onset SLE	HCQ, topical steroids, etoricoxib	Improvement of symptoms	[28]	
Sagy et al	23	M	1 month after 2nd dose	mRNA	Positive ANA, Ro/SSA, β2 Glycoprotein IgG, direct Coombs	New-onset SLE	HCQ, Prednisone, azathioprine, Granulocyte colony-stimulating factor, revolade	Improvement of symptoms	[28]	3
Sagy et al	56	M	1 month after 2nd dose	mRNA	Positive ANA, dsDNA, smith, low C3	New-onset SLE	HCQ, etoricoxib	Improvement of symptoms	[28]	
Molina-Rios et al.	42	F	2 weeks after 1st dose	mRNA	Positive ANA, anti-dsDNA Ab, low C4 level, IgG and IgM Beta-2-glycoprotein, elevated ESR and CRP, lupus anticoagulant	New-onset SLE	Azathioprine, HCQ, sulfasalazine, and pulse methylprednisolone with prednisolone taper	Improvement of symptoms	[29]	1
HidaKa et al.	53	F	Few days after 2nd dose	mRNA	Postive ANA, indirect and direct coombs, +lupus anticoagulant, thrombocytopenia, anemia,	New-onset SLE	Prednisolone with a taper	Improvement of symptoms	[30]	1
Zavala-Miranda et al.	23	F	1 week after 1st dose	Adenoviral vector	Positive ANA, elevated anti-dsDNA, low C3, C4	New-onset SLE, lupus nephritis	HCQ, MMF, high-dose	Improvement after 3 weeks	[31]	1

							corticosteroids			
Kim et al	60	F	1 month after 2nd dose	Adenoviral vector	Positive ANA, anti-dsDNA, anti-Sm antibody, low C3 and C4	New-onset SLE, lupus nephritis	Methylprednisolone and cyclophosphamide, followed by oral prednisolone and HCQ	Improvement of symptoms	[32]	1
Nelson et al.	14	M	2 days after 3rd dose	mRNA	Positive ANA, anti-dsDNA, anti-Sm, anti-RNP, anti-Ro, anti-La, hypocomplementemia	New-onset SLE, lupus nephritis	HCQ, prednisone, MMF	Improvement of symptoms	[33]	1
Salas et al.	NR	NR	After 1st dose	mRNA	NR	New-onset SLE, lupus nephritis	Steroids, hydroxychloroquine, and mycophenolate mofetil.	Improvement of symptoms	[34]	1

Table S2 Summary of case reports on RA after COVID-19 vaccination

Author	Age	Sex	Timeline of Symptom Onset	Type of Vaccine	Laboratory Features	Diagnosis	Treatment	Outcome	Ref	Case #
Matsuda et al.	79	F	2-3 day after the 2nd dose	mRNA	Elevated CRP, anti-CCP, RF	New onset RA	Biologics	Died	[25]	1
Yonezawa et al.	54	M	1 days after 2nd dose	mRNA	Elevated CRP, MMP-3, RF, ACPA, positive ANA	New onset RA	MTX and iguratimod	Improvement with treatment	[39]	1
Nahra et al.	74	M	10 days after the 1st dose	mRNA	Elevated ESR and CRP, RF	New-onset RA	Prednisone, Leflunomide	Symptom recurrence	[40]	1
Morikawa et al.	88	F	3 days after 1st dose	mRNA	Elevated CRP, anti-CCP, RF, and serum IL-6	New onset RA	Methylprednisolone	Improvement with treatment	[41]	1
Safary et al.	85	F	14 days after 1st dose	Inactivated virus	Elevated ESR and CRP, and positive RF and ANA	New onset RA	MTX, HCQ, and prednisolone	Improvement with treatment	[42]	2
Safary et al.	58	F	10 days after 1st dose	Adenoviral vector	Elevated ESR and CRP, and positive CCP and ACPA	New onset RA	MTX, HCQ, and prednisolone	Improvement with treatment	[42]	
Baimukhamedov et al.	38	F	20 days after 1st dose	Adenoviral vector	Elevated ESR and CRP, RF, positive ACPA	New onset RA	MTX, NSAIDs, methylprednisolone	Improvement with treatment	[43]	1
Watanabe et al.	53	M	4 weeks after 2nd dose	mRNA	Elevated CRP, anti-CCP, RF, leukocytosis	New onset RA	MTX, Tocilizumab	Complete Remission	[44]	1
Almouslem et al.	32	F	2 days after 2nd dose	mRNA	Elevated CRP, ANA, anti-RNP, anti-CCP, RF	New onset RA	Methylprednisolone, HCQ, prednisolone	Improvement with treatment	[45]	1

Table S3 Summary of case reports on VITT after COVID-19 vaccination

Author	Age	Sex	Timeline of Symptom Onset	Type of Vaccine	Laboratory Features	Diagnosis	Treatment	Outcome	Ref	Case #
Clerici et al.	≤39 y 130/333; 40–59 y 138/333 ; ≥60 y 65/333	Male, 153/332 (46%); Female, 179/332 (54%)	Median days (range); number of patients 9 (2–31); 207	Adenoviral vector	Elevated D-dimer, decreased platelet count, positive anti-PF4 antibodies	VITT	NR	Death, n/N (%): 69/290 (24)	[52]	366
Sistanizad et al.	18	Male	10 days after 1st dose	Inactivated	Elevated D-dimer, decreased platelet count	VITT	Apixaban, IVIG	Discharged home	[53]	1
Gabarin et al.	69	Male	1 week after 1st dose	Adenoviral vector	Thrombocytopenia, elevated fibrinogen, positive anti-PF4 antibody	Long VITT	High-dose IVIG, TPE, anticoagulants	NR	[54]	1
Roberge et al.	48	Female	16 days after vaccination	Adenoviral vector	Decreased platelet count, elevated D-dimer	VITT	IV heparin, argatroban, prednisone, IVIG, TPE	Discharged on rivaroxaban	[55]	2
Roberge et al.	56	Male	16 days after vaccination	Adenoviral vector	Elevated D-dimer, positive platelet-activation tests, thrombocytopenia	Long VITT	Prednisone, IVIG, argatroban and had a lower limb revascularization surgery, rivaroxaban	Discharged on rivaroxaban	[55]	
Roberge et al.	56	Male	16 days after vaccination	Adenoviral vector	Elevated D-dimer, positive platelet-		Prednisone, IVIG, argatroban	Improved	[56]	1

					activation tests, thrombocytopenia		and had a lower limb revascularization surgery, rivaroxaban			
Günther et al.	54	Male	12 days after 1st dose	Adenoviral vector	Elevated, D-dimer, positive anti-PF4 antibody, Thrombocytopenia	VITT	IVIG - Anticoagulation (argatroban, tinzaparin, danaparoid, fondaparinux) - Prednisolone	Died	[57]	1
Oliveira et al.	Median(Range), Total: 41(23–86), 38	Female, n/N (%):24/39 (61.5%): Male, n/N (%): 15/39 (38.5%)	Median(Range), Total: 8(0–37), 36	Adenoviral vector(97.5%); mRNA(2.5%)	Elevated D-dimer, decreased platelet count, positive anti-PF4 antibodies	VITT	IVIG, Plasma exchange, anticoagulation, glucocorticoids, rituximab	Death, n/N (%): 20/39 (51)	[58]	39
Lin et al.	28	Male	21 days after 3rd dose	mRNA	Elevated D-dimer, positive PF4 antibody assay	VITT	Methylprednisolone, IVIG, dabigatran	Fully recovered	[59]	1
Kataria et al.	28	Female	12 days after 2nd dose	Adenoviral vector	Elevated D-dimer, thrombocytopenia	VITT	Dexamethasone, IVIG, apixaban	Fully recovered	[60]	1
Chen et al.	42	Female	32 days after 3rd dose	mRNA	Elevated d-dimer, thrombocytopenia, anemia	VITT	Plasma exchange, IVIG, mechanical ventilation	Dead	[61]	1

Bellamy et al.	33	Male	12 days after a single dose	Adenoviral vector	Elevated d-dimer, thrombocytopenia, elevated prothrombin time, positive heparin-induced anti-PF4 antibodies	VITT	Levetiracetam, hemicraniectomy, thrombectomy, argatroban	Mechanical ventilation	[62]	1
Ali et al.	25	Female	5 days after 2nd dose	Adenoviral vector	Elevated d-dimers, thrombocytopenia, elevated c-reactive protein, thrombosis of the left transverse and superior sagittal sinus	VITT	Apixaban, IVIG	Fully recovered	[63]	1
Castan et al.	57	Male	10 days after a single dose	Adenoviral vector	Elevated D-dimer, thrombocytopenia, hepatic cytolysis, positive anti-PF4 antibodies	VITT	Intravenous acetylsalicylic acid and subcutaneous enoxaparin, apixaban	Improved	[64]	1
Edmonds et al.	43	Male	23 days after 1st dose	Adenoviral vector	Elevated D-dimer, decreased platelet count, increased fibrinogen, positive anti-PF4/heparin IgG	VITT	Argatroban, IVIG	Brain dead	[65]	1

Yelne et al.	25	Male	17 days after 1st dose	Adenoviral vector	Elevated D-dimer, positive anti-PF4 antibody	VITT	Heparin, levetiracetam, rivaroxaban	Fully recovered	[66]	1
Hung et al.	49	Male	21 days after 1st dose	Adenoviral vector	Elevated D-dimer, positive anti-PF4 antibodies	VITT	Coronary artery bypass surgery, forefoot amputation	Partial improvement	[67]	1
Jain et al.	18	Male	8 days after 1st dose	Adenoviral vector	Elevated D-dimer, positive anti-PF4 autoantibody	VITT	Prednisolone, fondaparinux, resection and anastomosis of the affected small bowel, platelet transfusion, IVIG, apixaban	Fully recovered	[68]	1
Ge et al.	46	Female	11 days after 1st dose	Adenoviral vector	Elevated D-dimer, thrombocytopenia	VITT	Nonheparin AC, IVIG, steroids	Recovered	[69]	4
Ge et al.	46	Female	17 days after 1st dose	Adenoviral vector	Elevated D-dimer, thrombocytopenia	VITT	Nonheparin AC, IVIG, steroids	Recovered	[69]	
Ge et al.	46	Female	10 days after 1st dose	Adenoviral vector	Elevated D-dimer, venous or arterial thrombosis	VITT	Nonheparin AC, IVIG, steroids, therapeutic plasma exchange	Recovered	[69]	

Ge et al.	55	Male	31 days after 1st dose	Adenoviral vector	Elevated D-dimer, thrombocytopenia, deep vein thrombosis	VITT	Argatroban	Recovered	[69]	
Comolli et al.	75	Male	12 hours after 1st dose	Adenoviral vector	Elevated D-dimer, thrombocytopenia, positive anti-PF4 antibodies	VITT	Not reported	Not reported	[70]	1
Ling et al.	27	Female	7 days after 3rd dose	mRNA	Elevated D-dimer, thrombocytopenia, positive anti-PF4 antibodies	VITT	IVIG, prednisolone, rivaroxaban	Fully recovered	[71]	1
Palmer et al.	59	Female	19 days after 1st dose	Adenoviral vector	Elevated D-dimer, thrombocytopenia, positive anti-PF4 antibodies	VITT	IVIg, Dexamethasone, Argatroban IVI, Surgical intervention, Aspirin and rivaroxaban postoperatively	Improved	[72]	2
Palmer et al.	58	Male	11 days after 1st dose	Adenoviral vector	Elevated D-dimer, thrombocytopenia, elevated glucose, elevated CRP	VITT	IVIg, Fondaparinux, 6 months rivaroxaban	Recovered	[72]	
Doubrovinskaia et al.	43	Female	11 days after 1st dose	Adenoviral vector	PF4-Ab positive	VITT	UFH, LMWH, IVIGs, dexa, apixaban		[73]	3

Doubrovinskaia et al.	27	Female	12 days after 1st dose	Adenoviral vector	PF4-Ab positive	VITT	Argatroban, IVIGs, dexa, dabigatran		[73]	
Doubrovinskaia et al.	25	Female	14 days after 1st dose	Adenoviral vector	NR	VITT	Argatroban, dabigatran		[73]	
Savulescu et al.	60	Female	14 days after a single dose	Adenoviral vector	Elevated, D-dimer, glucose, CRP. Decreased PLT. Extensive thrombosis.	VITT	Extensive bowel resection with end jejunostomy and feeding ileostomy, heparin	Improved	[74]	1
Zaheri et al.	46	Female	3 days after 1st dose	Inactivated	Elevated, D-dimer, positive anti-PF4 antibody. Decreased PLT. Thrombocytopenia	VITT	Dabigatran, IVIG, aspirin	Discharged in stable condition	[75]	1

Table S4 Summary of case reports on GBS after COVID-19 vaccination

Author	Age	Sex	Timeline of Symptom Onset	Type of Vaccine	Laboratory Features	Diagnosis	Treatment	Outcome	Ref	Case #
McKean et al.	48	M	10 days after 1st dose	Adenoviral vector	High protein level and lymphocytosis in CSF.	New-onset GBS	IVIg, oral prednisolone	Improved	[82]	1
Chun et al.	80	F	1 week after 2nd dose	mRNA	Axonal type sensorimotor peripheral polyneuropathy, mild-to-moderate irregular increased 18F-FDG uptake	New-onset GBS	High-dose methylprednisolone, IVIg	Improved slightly, referred to rehabilitation facility	[83]	2
Chun et al.	76	F	4 weeks after 2nd dose	mRNA	Sensorimotor peripheral polyneuropathy, CSF analysis showed cytoalbuminologic dissociation	New-onset GBS	IVIg	Proximal weakness and tingling relieved, referred to rehabilitation facility	[83]	
Hasan et al.	62	F	11 days after 1st dose	Adenoviral vector	Albuminocytological dissociation with elevated protein and WBC count.	New-onset GBS	IVIg	Deteriorated, mechanical ventilation	[84]	1
García-Grimshaw et al.	33	M	28 days after 1st dose	mRNA	Elevated CSF Proteins, normal WBC,	New-onset GBS	IVIg	Discharged home	[85]	7

					elevated CSF glucose				
García-Grimshaw et al.	25	M	12 days after 1st dose	mRNA	Elevated CSF Proteins, elevated WBC, decreased CSF glucose	New-onset GBS	IVIg	Discharged home	[85]
García-Grimshaw et al.	53	F	6 days after 1st dose	mRNA	Decreased CSF Proteins, normal WBC, elevated CSF glucose	New-onset GBS	IVIg	Hospitalized	[85]
García-Grimshaw et al.	72	M	4 days after 1st dose	mRNA	Not performed	New-onset GBS	IVIg	Discharged home	[85]
García-Grimshaw et al.	31	M	11 days after 1st dose	mRNA	Not performed	New-onset GBS	IVIg	Discharged home	[85]
García-Grimshaw et al.	67	F	4 days after 1st dose	mRNA	Normal WBC, elevated protein, and glucose in CSF.	New-onset GBS	IVIg	Died	[85]
García-Grimshaw et al.	81	F	3 days after 1st dose	mRNA	Elevated WBC, protein, and glucose in CSF.	New-onset GBS	IVIg	Discharged home	[85]
Castiglione et al.	56	F	19 days after 1st dose	Adenoviral vector	Mild elevation in CSF protein with very low white cell count	New-onset GBS, BFP variant	IVIg	Improved	[86]
Castiglione et al.	55	M	28 days after 1st dose	Adenoviral vector	Markedly elevated CSF protein with low white cell count,	New-onset GBS, BFP variant	IVIg	Improved	[86]

					indicative of a significant inflammatory or other pathological process.				
Castiglione et al.	87	M	17 days after 1st dose	Adenoviral vector	Elevated CSF protein with a slightly higher white cell count, GD1a positive	New-onset GBS, BFP variant	None	Sudden arrhythmia death	[86]	
Castiglione et al.	50	M	20 days after 1st dose	Adenoviral vector	Slightly elevated CSF protein with no white cells	New-onset GBS, BFP variant	IVIg	Improved	[86]	
Castiglione et al.	39	M	10 days after 1st dose	Adenoviral vector	Sulfatide positive	New-onset GBS, BFP variant	IVIg	Improved	[86]	
Castiglione et al.	42	M	28 days after 1st dose	Adenoviral vector	Slightly elevated CSF protein with no white cells	New-onset GBS, BFP variant	IVIg	Improved	[86]	
Castiglione et al.	52	F	13 days after 1st dose	Adenoviral vector	Mildly elevated CSF protein with no white cells, GM1 positive	New-onset GBS, BFP variant	Plasma Exchange	Improved	[86]	
Castiglione et al.	43	M	13 days after 1st dose	Adenoviral vector	Slightly elevated CSF protein with a higher white cell count	New-onset GBS, BFP variant	IVIg	Improved	[86]	
Castiglione et al.	65	M	7 days after 1st dose	Adenoviral vector	Slightly elevated CSF protein with a low white cell	New-onset GBS, BFP variant	IVIg	Improved	[86]	

					count, GM1 positive					
Germano et al.	68	M	12 days after 1st dose	Adenoviral vector	Normal WBC, low protein, and high glucose in CSF.	New-onset GBS	IVIg	Recovered	[87]	13
Germano et al.	71	F	10 days after 1st dose	mRNA	Not performed.	New-onset GBS	IVIg	Improved	[87]	
Germano et al.	40	F	4 days after the 2nd dose	mRNA	Not performed.	New-onset GBS	IVIg	Recovered	[87]	
Germano et al.	89	M	15 days after 1st dose	mRNA	Elevated WBC, protein, and glucose in CSF.	New-onset GBS	IVIg	Recovered	[87]	
Germano et al.	65	M	15 days after 1st dose	Adenoviral vector	Elevated protein and glucose in CSF.	New-onset GBS	plasma exchange	Further IVIg	[87]	
Germano et al.	80	M	21 days after 2nd dose	mRNA	high CSF protein, high lymphomonocyte counts	New-onset GBS	IVIg	Recovered	[87]	
Germano et al.	69	F	17 days after 1st dose	Adenoviral vector	NR	New-onset GBS	IVIg	Further IVIg	[87]	
Germano et al.	18	M	12 days after 1st dose	mRNA	NR	New-onset GBS	IVIg	Recovered	[87]	
Germano et al.	57	M	5 days after 1st dose	mRNA	high CSF protein	New-onset GBS	IVIg	Recovered	[87]	
Germano et al.	64	M	15 days after 1st dose	Adenoviral vector	not performed	New-onset GBS	IVIg	Recovered	[87]	
Germano et al.	88	M	15 days after 2nd dose	mRNA	high CSF protein	New-onset GBS	IVIg	Recovered	[87]	
Germano et al.	73	F	5 days after 1st dose	Adenoviral vector	not performed	New-onset GBS	None	Recovered	[87]	
Germano et al.	51	M	4 days after 1st dose	mRNA	NR	New-onset GBS	None	Recovered	[87]	

Waheed et al.	82	F	1 week after 1st dose	mRNA	Albuminocytologic dissociation with high protein and low WBC count.	New-onset GBS	IVIg	Improved	[88]	1
Razok et al.	73	M	20 days after 2nd dose	mRNA	Elevated protein and albumin in CSF.	New-onset GBS	IVIg	Improved	[89]	1
Patel et al.	37	M	2 weeks after 1st dose	Adenoviral vector	Elevated protein in CSF.	New-onset GBS	IVIg	Slow improvement	[90]	1
Allen et al.	54	M	16 days after 1st dose	Adenoviral vector	Mild lymphocytosis and elevated protein in CSF.	New-onset GBS	oral prednisolone	Improved	[91]	4
Allen et al.	20	M	26 days after 1st dose	Adenoviral vector	Mild lymphocytosis and elevated protein in CSF.	New-onset GBS	oral prednisolone	Improved	[91]	
Allen et al.	57	M	21 days after 1st dose	Adenoviral vector	Mild lymphocytosis and elevated protein in CSF.	New-onset GBS	IVIg	Stable	[91]	
Allen et al.	55	M	29 days after 1st dose	Adenoviral vector	MRI enhancement of facial nerve; normal cell count, elevated protein in CSF.	New-onset GBS	No treatment	Improved	[91]	

Maramattom et al.	43	F	10 days after 1st dose	Adenoviral vector	a demyelinating neuropathy and high CSF protein	New-onset GBS	IVIg, IMV	Recovered	[92]	4
Maramattom et al.	67	F	14 days after 1st dose	Adenoviral vector	a demyelinating neuropathy and high CSF protein	New-onset GBS	IVIg, IMV, Plasmapheresis	Improved	[92]	
Maramattom et al.	53	F	12 days after 1st dose	Adenoviral vector	a demyelinating neuropathy and high CSF protein	New-onset GBS	IVIg, IMV	Mechanical ventilation	[92]	
Maramattom et al.	68	F	14 days after 1st dose	Adenoviral vector	a demyelinating neuropathy and high CSF protein	New-onset GBS	IVIg, IMV	Mechanical ventilation	[92]	
Sosa-Hernández et al.	23	M	1 day after 2nd dose	mRNA	Symmetrical motor demyelinating polyradiculoneuropathy on EMG.	New-onset GBS	IVIg and steroid therapy	Improved	[93]	1
Fakhari et al.	60	M	20 days after 3rd dose	Inactivated Virus	Low amplitude sensory response and reduced motor response in lower limb.	New-onset GBS	IVIg	Improved	[94]	1
Rao et al.	42	F	7 days after 2nd dose	mRNA	Elevated CSF protein, normal WBC count.	New-onset GBS	IVIg	Improved	[95]	1

Bellucci et al.	57	M	5 days after 1st dose	mRNA	Albuminocytological dissociation in CSF, demyelinating sensory-motor polyneuropathy on EMG.	New-onset GBS	IVIg	Recovered	[96]	1
Masuccio et al.	NR	M	45 days after 1st dose	mRNA	Albumin-cytological dissociation in CSF, mixed axonal and demyelinating features on EMG.	New-onset GBS	IVIg	Improved	[97]	1
Kripalani et al.	52	F	9 days after 1st dose	Adenoviral vector	Elevated CSF protein, normal leucocyte count, mild demyelinating distal motor neuropathy.	New-onset GBS	IVIg	Hospitalized	[98]	1
Thant et al.	66	M	14 days after a single dose	Adenoviral vector	Elevated CSF protein, albumin-cytological dissociation.	New-onset GBS	IVIg, plasma exchange	Hospitalized	[99]	1
Silva et al.	62	F	18 days after 1st dose	Adenoviral vector	Elevated CSF protein, normal glucose, low WBC count.	New-onset GBS	IVIg	Improved	[100]	1
Wan et al.	40	M	10 days after 1st dose	Adenoviral vector	Absent F-waves in nerve conduction	New-onset GBS	IVIg	Improved	[101]	3

					studies, demyelinating polyneuropathy.				
Wan et al.	53	F	12 days after 1st dose	Adenoviral vector	Demyelinating polyneuropathy on EMG	New-onset GBS	plasma exchange	Hospitalized	[101]	
Wan et al.	59	M	14 days after 1st dose	Adenoviral vector	Elevated protein in CSF	New-onset GBS	IVIg	Improved	[101]	
Čenščák et al.	42	M	14 days after 1st dose	mRNA	Albuminocytological dissociation in CSF, enhancement in LS spine MRI.	New-onset GBS	IVIg	Improved	[102]	1
Oo et al.	51	M	14 days after 1st dose	Adenoviral vector	Albuminocytologic dissociation in CSF, demyelinating pathology of GBS on NCS.	New-onset GBS	IVIg	Improved	[103]	4
Oo et al.	65	F	14 days after 1st dose	Adenoviral vector	Albuminocytologic dissociation in CSF, negative viral panel, demyelinating polyneuropathy.	New-onset GBS	IVIg	Improved	[103]	
Oo et al.	72	M	21 days after 1st dose	Adenoviral vector	Albuminocytologic dissociation in CSF, prolonged distal motor	New-onset GBS	IVIg	Recovered	[103]	

					latency on NCS.					
Oo et al.	66	M	21 days after 1st dose	Adenoviral vector	Albuminocytologic dissociation in CSF, demyelinating nature of GBS on NCS.	New-onset GBS	IVIg	Improved	[103]	
Scendoni et al.	82	F	14 days after 1st dose	mRNA	Albumin cytologic dissociation in CSF, demyelinating pathology of GBS on NCS.	New-onset GBS	IVIg	Improved	[104]	1
Sriwastava et al.	67	F	1 day after 1st dose	mRNA	Normal CSF chemistry, cell count, and absent oligoclonal bands.	New-onset GBS	IVIg and plasmapheresis	Improved	[105]	3
Sriwastava et al.	41	M	14 days after a single dose	Adenoviral vector	Increased protein, normal cell count, albuminocytological dissociation in CSF.	New-onset GBS	IVIg	Improved	[105]	
Sriwastava et al.	42	M	60 days after 2nd dose	mRNA	Increased protein in CSF, orbital MRI findings.	New-onset GBS	IV solumedrol and oral prednisone	Improved	[105]	
Suri et al.	47	M	17 days after 1st dose	Adenoviral vector	Acute inflammatory demyelinating polyradiculone	New-onset GBS	IVIg and oral prednisolone and azathioprine	Improved	[106]	1

					uropathy on NCV.					
Introna et al.	62	M	10 days after 1st dose	Adenoviral vector	Albumin-cytologic dissociation in CSF, high opening pressure.	New-onset GBS	IVIg	Improved	[107]	1
Bouattour et al.	67	M	7 days after 1st dose	mRNA	Albuminocytological dissociation in CSF, normoglucorrachia, demyelinating polyneuropathy.	New-onset GBS	IVIg	Recovered	[108]	1
Min et al.	58	M	3 days after 1st dose	Adenoviral vector	Albuminocytologic dissociation in CSF, demyelinating sensory-motor polyneuropathy.	New-onset GBS	Gabapentin	Improved	[109]	2
Min et al.	37	F	4 days after 1st dose	Adenoviral vector	Decrease in distal leg IENFD, small fiber neuropathy.	New-onset GBS	Gabapentin, Duloxetine, and Tramadol	Improved	[109]	
Nasuelli et al.	59	M	10 days after 1st dose	Adenoviral vector	Elevated CSF protein, normal WBC count, glycorrhachia, motor	New-onset GBS	IVIg	Improved	[110]	1

					polyradiculoneuropathy.					
Morehouse et al.	49	F	5 days after a single dose	Adenoviral vector	Small punctate foci on MRI, equivocal B12 level.	New-onset GBS	B12 injection, IVIG, Plasmapheresis	Improved	[111]	1
Andreozzi et al.	59	F	15 day after 1st dose	Adenoviral vector	Normal CSF opening pressure, glucose, proteins, and WBC count.	New-onset GBS, AIDP variant	IVIg	Recovered	[112]	2
Andreozzi et al.	43	M	7 days after 1st dose	Adenoviral vector	Normal CSF opening pressure, glucose, proteins, and WBC count.	New-onset GBS, AIDP variant	IVIg	Recovered	[112]	
Tabatabaee et al.	46	M	3 days after 1st dose	Adenoviral vector	High protein in CSF, acute motor axonal neuropathy on EMG.	New-onset GBS	IVIg	Partial improved	[113]	3
Tabatabaee et al.	36	M	5 days after 1st dose	Inactivated Virus	Slight elevation of protein in CSF, AMAN form of GBS on EMG.	New-onset GBS, AMAN variant	IVIg	Partial improved	[113]	
Tabatabaee et al.	32	M	14 days after 1st dose	Inactivated Virus	Normal protein level in CSF, AMAN variant of GBS on EMG.	New-onset GBS, AMAN variant	IVIg	Partial improved	[113]	
Hughes et al.	65	M	2 days after 1st dose	mRNA	Elevated protein in CSF, normal	New-onset GBS, AIDP variant	IVIg	Improved	[114]	1

					cell count, demyelinating polyneuropathy on EMG.					
Rohilla et al.	13	F	4 days after 1st dose	Protein Subunit	Pure motor axonal polyneuropathy on NCV.	New-onset GBS	plasmapheresis	Improved	[115]	1
Biswas et al.	49	M	7 days after 1st dose	Adenoviral vector	Demyelinating involvement of upper and lower limbs on NCS.	New-onset GBS	IVI and injection Prednisolone	Improved	[116]	1
Dalwadi et al.	86	F	2 days after 2nd dose	mRNA	Albuminocytological dissociation in CSF, demyelinating polyneuropathy on NCS.	New-onset axonal-variant GBS	plasmapheresis	No notable improvement	[117]	1
Rossetti et al.	38	M	14 days after a single dose	Adenoviral vector	Elevated ferritin, CRP, thrombocytosis, detected albuminocytological dissociation	New-onset atypical variant of GBS	IVIg	Improved	[118]	1
Lanman et al.	58	F	3 days after 1st dose	mRNA	Mixed demyelinating and axonal features on EMG, demyelinating polyneuropathy.	New-onset GBS	IVIg, prednisone	Improved	[119]	1
Dang et al.	63	M	9 days after 1st dose	Adenoviral vector	Albuminocytologic dissociation in	New-onset GBS, MFS	IVIg	Improved	[120]	1

					CSF, long-standing axonal neuropathy on EMG.					
Kim et al.	16	F	2 days after 2nd dose	mRNA	Mild thickening and enhancement of spinal nerve roots on MRI, elevated protein in CSF.	New-onset GBS	continued follow-up with neurology and physical therapy treatment	Improved	[121]	1
Ogata et al.	70	M	2 days after 2nd dose	mRNA	Albuminocytological dissociation in CSF, demyelinating neuropathy on NCS.	New-onset GBS	IVIg, methylprednisolone	Improved	[122]	1
Nagalli et al.	49	F	10 days after 1st dose	mRNA	Albuminocytological dissociation in CSF.	“sub-acute” onset of GBS	Plasma exchange	Improved	[123]	1
Aldeeb et al.	81	F	14 days after 1st dose	mRNA	Demyelinating polyneuropathy with secondary axonal degeneration on EMG.	New-onset GBS	IVIg	Fully recovered	[124]	1
Kim et al.	42	M	22 days after 1st dose	Adenoviral vector	Albumin-cytologic dissociation in CSF, early axonal-type polyneuropathy on EMG.	New-onset GBS	IVIg with plasma exchange	Partial improved	[125]	2

Kim et al.	48	F	14 days after 1st dose	mRNA	Elevated protein, high albumin, normal cell count, motor-dominant mixed polyneuropathy.	New-onset GBS	IVIg	Fully recovered	[125]	
Zubair et al.	30	F	14 days after a single dose	Adenoviral vector	Zero nucleated cells, elevated protein, normal glucose in CSF.	New-onset Facial Diplegia Variant of GBS in Pregnancy	IVIg	Fully recovered	[126]	1
Silfat et al.	67	M	14 days after 1st dose	Adenoviral vector	Albumino-cytological dissociation in CSF, negative infection screen.	New-onset GBS	IVIg	Improved	[127]	1
Chang et al.	48	F	14 days after 1st dose	Adenoviral vector	Albumin-cytological dissociation in CSF, severe demyelination on EMG.	New-onset GBS	IVIg, gabapentin and prednisolone	Improved	[128]	1
Prasad et al.	41	M	12 days after a single dose	Adenoviral vector	Elevated WBC, protein in CSF, demyelinating GBS on EMG.	New-onset GBS	IVIg	Be placed in a rehabilitation facility	[129]	1
Kanabar et al.	61	F	10 days after 1st dose	Adenoviral vector	Elevated protein, normal cell count, demyelinating	New-onset GBS	IVIg	Improved	[130]	2

					polyneuropath y on NCS.					
Kanabar et al.	56	M	7 days after 1st dose	Adenoviral vector	Elevated protein, normal cell count, demyelinating polyneuropath y on NCS.	New-onset GBS	IVIg	Complete recovery	[130]	
Hilts et al.	58	M	3 days after 1st dose	mRNA	Elevated protein in CSF, normal WBC count.	New-onset GBS	IVIg followed by plasmapheresis	Moderate improvement	[131]	1
Hai et al.	38	M	4 days after 1st dose	Adenoviral vector	Albuminocytologic dissociation in CSF, demyelinating pathology of GBS on NCS.	New-onset GBS	plasmapheresis	Improved	[132]	2
Hai et al.	29	M	21 days after 2nd dose	Adenoviral vector	Albuminocytologic dissociation in CSF, absent pleocytosis.	New-onset GBS	plasmapheresis	Improved	[132]	
Siddiqi et al.	53	M	8 days after 1st dose	Inactivated Virus	Elevated proteins, no pleocytosis; Prolonged latencies, reduced conduction velocities, prolonged F waves	New-onset GBS, MFS	regular physiotherapy	Fully recovered	[133]	1

Shalash et al.	39	M	5 days after 2nd dose	mRNA	NCV revealed sensory and motor axonal neuropathy.	New-onset GBS	plasmapheresis, intravenous methylprednisolone	Complete improvement	[134]	
Shalash et al.	29	M	20 days after 2nd dose	Adenoviral vector	Axonal polyneuropathy of both lower limbs, proximal neurogenic affection	New-onset GBS	plasmapheresis, intravenous methylprednisolone	Partial improvement	[134]	
Shalash et al.	59	M	30 days after 2nd dose	Adenoviral vector	Bilateral axonal polyradiculoneuropathy of both lower limbs	New-onset GBS	plasmapheresis, intravenous methylprednisolone	Complete improvement except residual numbness	[134]	
Shalash et al.	53	M	14 days after 2nd dose	Adenoviral vector	Bilateral axonal polyradiculoneuropathy of LLs.	New-onset GBS	plasmapheresis, intravenous methylprednisolone	Partial improvement	[134]	7
Shalash et al.	55	M	30 days after 2nd dose	mRNA	Bilateral axonal polyradiculoneuropathy of LLs.	New-onset GBS	plasmapheresis, intravenous methylprednisolone	Complete improvement	[134]	
Shalash et al.	59	M	30 days after 3rd dose	Inactivated Virus	Elevated protein in CSF	New-onset GBS	plasmapheresis	Complete improvement	[134]	
Shalash et al.	29	F	60 days after 2nd dose	Adenoviral vector	Mixed axonal and demyelinating polyradiculoneuropathy	New-onset GBS	plasmapheresis, intravenous methylprednisolone, prednisone	Partial improvement	[134]	

Carranza et al.	53	F	14 days after a single dose	Adenoviral vector	mild elevated protein, slightly elevated mononuclear pleocytosis	New-onset GBS	IVIg	Complete improvement	[135]	1
Bazrafsha et al.	68	F	3 days after 2nd dose	Adenoviral vector	Elevated protein in CSF	New-onset GBS, AIDP variant	IVIg	Complete improvement	[136]	1
Prado et al.	35	M	10 days after 2nd dose	Inactivated Virus	Preserved CMAP, SNAP, motor and sensory NCV, F-wave latencies	New-onset GBS	acyclovir, prednisone, facial muscle physical therapy	Marked improvement	[137]	1
Donaldson et al.	45	M	12 days after 1st dose	Adenoviral vector	Elevated CSF protein and white blood cell count, demyelinating polyradiculopathy	New-onset GBS	IVIg	Improved	[138]	1
Wimmer Del-Solar et al.	50	F	2 days after 1st dose	Inactivated Virus	Axonal motor polyneuropathy	New-onset GBS, AMAN variant	IVIg	Fully recovered	[139]	1
Lázaro et al.	60	M	9 days after 1st dose	mRNA	Sensorimotor polyradiculoneuropathy, demyelinating, absence of sensory potentials	New-onset GBS	IVIg	Fully recovered	[140]	1
Abičić et al.	24	F	18 days after 1st dose	mRNA	Albuminocytological dissociation	New-onset GBS, MFS	IVIg	partial improvement	[141]	1

George et al.	60	M	28 days after 1st dose	mRNA	Albuminocytological dissociation	New-onset GBS	IVIg	Complete recovery	[142]	1
Katada et al.	44	F	2 days after 2nd dose	mRNA	Marked demyelinating sensorimotor polyneuropathy, prolonged F wave latencies	New-onset GBS, CIDP	IVIg	partial improvement	[143]	1
Hwang et al.	47	M	28 days after 1st dose	mRNA	Elevated protein, WBC count, and decreased glucose levels in CSF	New-onset GBS	IVIg	fully recovered	[144]	1
Oshibe et al.	71	F	7 days after 1st dose	mRNA	CSF: Albumino cytological dissociation. NCS: functional axonal disturbance.	New-onset GBS	IVIg	fully recovered	[145]	1
Kim et al.	21	M	21 days after 1st dose	mRNA	Albuminocytologic dissociation, elevated protein; Sural sparing pattern, mild prolongation of F waves, decreased CMAPs	New-onset GBS	IVIg	mild recovery	[146]	1
Nanatsue et al.	70	M	7 days after 2nd dose	mRNA	Nerve conduction studies revealed F-	New-onset GBS, MFS	IVIg,Steroids, valacyclovir, and	fully recovered	[147]	1

					wave abnormalities.		mecobalamin			
Pirola et al.	47	F	7 days after 1st dose	Adenoviral vector	Albuminocytological dissociation, elevated protein in CSF	New-onset GBS, MFS	IVIG, gabapentin	fully recovered	[148]	1
Chen et al.	70	F	14 days after 2nd dose	mRNA	Localized neuropathy affecting the left facial nerve and motor nerves supplying the lower leg muscles.	New-onset GBS, MFS	IVIg	Improved	[149]	1
Algahtani et al.	46	F	5 days after 1st dose	mRNA	CSF showed one white blood cell with a high protein concentration of 1.39 g/L. Nerve conduction study of the four extremities reported acute demyelinating sensory and motor polyneuropathy with prolonged F-wave latencies.	New-onset GBS	IVIg	fully recovered	[150]	1

Acharya et al.	78	M	4 days after 2nd dose	Adenoviral vector	Albuminocytological dissociation.	New-onset GBS, AIDP variant	IVIg	fully recovered	[151]	1
Do et al.	59	M	60 days after 2nd dose	mRNA	Nerve conduction and electromyography studies revealed demyelination and dysfunctions in the engagement of distal muscles below the knee	GBS/TM overlap syndrome	IVIg	Improved	[152]	1
Wijekoon et al.	41	M	18 days after 1st dose	Inactivated Virus	Cell protein dissociation, elevated CSF protein; Reduced motor nerve conduction velocities, prolonged distal motor latencies	New-onset GBS	IVIg	fully recovered	[153]	1
Oliveira et al.	63	M	14 days after 1st dose	Adenoviral vector	Albuminocytologic dissociation, elevated protein; Severe acquired acute demyelinating polyneuropathy	New-onset GBS	IVIg followed by plasmapheresis	slight improvement	[154]	1

Hurtado et al.	29	M	9 days after 1st dose	Adenoviral vector	Motor demyelination polyneuropathy	New-onset GBS	plasmapheresis	fully recovered	[155]	1
Berrim et al.	41	F	7 days after 2nd dose	Adenoviral vector	Albuminocytological dissociation, elevated protein; Demyelinating pathology of GBS	New-onset GBS	IVIg	Improved	[156]	4
Berrim et al.	53	M	7 days after 1st dose	Adenoviral vector	Slightly elevated CSF protein; Mild demyelinating distal motor neuropathy	New-onset GBS	IVIg	fully recovered	[156]	
Berrim et al.	80	M	28 days after a single dose	Adenoviral vector	Typical features of AIDP	New-onset GBS	IVIg	Relapse after 2 months	[156]	
Berrim et al.	62	M	21 days after 3rd dose	mRNA	Diffuse motor demyelinating polyneuropathy	New-onset GBS	IVIg	fully recovered	[156]	
Lee et al.	43	F	9 days after 1st dose	Adenoviral vector	Lumbosacral polyradiculopathy	New-onset GBS	plasmapheresis	Improved	[157]	2
Lee et al.	54	M	12 days after 1st dose	Adenoviral vector	Demyelinating polyradiculoneuropathy, bilateral trigeminal neuropathy	New-onset GBS	IVIg	Improved	[157]	
Lee et al.	32	M	7 days after 1st dose	Adenoviral vector	Elevated CSF protein, no cells; Acute, severe,	New-onset GBS, AMSAN variants	IVIg	Improved	[158]	1

					diffuse, sensorimotor polyradiculoneuropathy				
Sii et al.	53	F	8 days after 1st dose	Inactivated Virus	Albuminocytologic dissociation, elevated protein, elevated glucose; Reduced ulnar SNAP, normal sural SNAP	New-onset GBS	IVIg	gradually recovered	[159]	1
Zhu et al.	58	M	55 days after 2nd dose	NR	High protein level, leukocytes; Severe multifocal sensorimotor demyelinating polyneuropathy	New-onset GBS	immunoglobulin treatment, plasma exchange, methylprednisolone, and rehabilitation training	Improved	[160]	1
Duong-Quy et al.	22	M	14 days after 1st dose	Inactivated Virus	Cytoalbuminologic dissociation	New-onset GBS	dexamethasone, cefadroxil and levofloxacin	fully recovered	[161]	1
Soh et al.	49	M	10 days after 1st dose	Adenoviral vector	Albuminocytologic dissociation; Moderate to severe sensory-motor axonal polyneuropathy	New-onset GBS	IVIg with plasma exchange	Improved	[162]	1

Kamar et al.	16	M	8 days after 1st dose	mRNA	Albumin-cytological dissociation	New-onset GBS	IVIg	Improved	[163]	1
Smaoui et al.	41	M	15 day after 1st dose	mRNA	Albumin-cytological dissociation; Typical features of AIDP	New-onset GBS	IVIg	Improved	[164]	1
Sukockienė et al.	30	M	15-20 days after 1st dose	Adenoviral vector	Contrast enhancement on both facial nerves and sacral roots	New-onset GBS	IVIg	Improved	[165]	2
Sukockienė et al.	90	F	21 days after 1st dose	mRNA	NR	New-onset GBS	Spontaneous recovery without treatment	Improved	[165]	

Table S5 Summary of case reports on AIH after COVID-19 vaccination

Author	Age	Sex	Timeline of Symptom Onset	Type of Vaccine	Laboratory Features	Diagnosis	Treatment	Outcome	Ref	Case#
Clayton-Chubb et al.	36	M	26 days after 1st dose	Adenoviral vector	Elevated ALT, AST, GGT, bilirubin, positive ANA antibody	New-onset AIH	Prednisolone	Improvement with treatment	[170]	1
Mekritthikrai et al.	52	F	7 days after 2nd dose	Inactivated	Elevated AST, ALT, ALP, bilirubin, positive ANA, ASMA, high IgG, liver biopsy showing lymphoplasmacytic infiltration, interface hepatitis, periportal bridging fibrosis	New-onset AIH	Prednisolone, Azathioprine	Improvement with treatment	[171]	1
Bril et al.	35	F	6 days after 1st dose	mRNA	Elevated bilirubin, AST, ALT, alkaline phosphatase, positive ANA, positive double-stranded DNA antibodies	New-onset AIH	Prednisone	Improved symptoms, normalized liver enzymes	[172]	1
Rela et al.	38	F	2 weeks after 1st dose	Adenoviral vector	Elevated bilirubin, AST, ALT, positive ANA, Elevated IgG, Elevated anti-SARS-	New-onset AIH	Prednisolone	Improved symptoms, normalized liver enzymes	[173]	2

					CoV2 spike protein antibody					
Rela et al.	62	M	16 days after 1st dose	Adenoviral vector	Elevated bilirubin, AST, ALT, Elevated anti-SARS-CoV2 spike protein antibody	New-onset AIH	Prednisolone, therapeutic plasma exchange	Death after three weeks of admission	[173]	
Mathew et al.	Late 20s	F	10 days after first dose	Adenoviral vector	Elevated AST, ALT, total bilirubin, direct bilirubin, total protein, ANA, IgG, IgM	New-onset AIH	Ursodeoxycholic acid, pantoprazole, S-adenosyl-L-methionine, N-acetylcysteine, vitamin K4, prednisolone, cholecalciferol, esomeprazole, calcium carbonate+vitamin D3, azathioprine, multivitamin capsule	Improved	[174]	1
Romero-Salazar et al.	76	M	After third dose	mRNA	Elevated ALT, AST, bilirubin, IgG, ANA, hepatitis, necrosis, inflammation	New-onset AIH	Steroids, azathioprine	Improved	[175]	1

Ruiz et al.	51	F	14 days after 2nd dose	mRNA	Elevated bilirubin, GOT, GPT, ANA, SMA	New-onset AIH	Corticosteroids, azathioprine	Improvement in liver enzymes and symptoms, no relapse reported	[176]	3
Ruiz et al.	80	M	12 days after 1st dose	mRNA	Elevated bilirubin, GOT, GPT, ANA, SMA	New-onset AIH	Corticosteroids (low dose maintenance therapy)	Improvement in liver enzymes and symptoms, no relapse reported	[176]	
Ruiz et al.	56	M	22 days after 3rd dose	mRNA	Elevated bilirubin, GOT, GPT, SMA	New-onset AIH	Corticosteroids, azathioprine	Improvement in liver enzymes and symptoms, no relapse reported	[176]	
Yoshida et al.	85	F	8 weeks after 2nd dose	mRNA	Elevated AST, ALT, γ-GT, total bilirubin, γ-globulin, IgG, ANA, rheumatoid factor	New-onset AIH	Prednisolone, aspirin (stopped prior to biopsy)	Liver function normalized	[177]	1
Gips et al.	72	M	2 weeks after 1st dose	mRNA	Elevated WBC, AST, ALT, total bilirubin, ALP, iron, ferritin, IgG, ANA, ASMA	New-onset AIH	Prednisone, mycophenolate mofetil	Complete normalization of liver enzymes	[178]	1

Zafar et al.	51	M	After each dose	Adenoviral vector	Elevated bilirubin, ALT, ALP, CRP	New-onset AIH	Ursodeoxycholic acid, S-adenosyl-L-methionine, N-acetylcysteine, vitamin K, prednisolone, cholecalciferol, esomeprazole, calcium carbonate + vitamin D3, azathioprine	liver function normalized with conservative treatment	[179]	2
Zafar et al.	81	F	After 4th dose	mRNA	Elevated ALT, bilirubin, ALP, globulins, ferritin, CRP; Decreased albumin, total proteins	New-onset AIH	COVID-19 Vaccination-Induced Cholangiopathy and AIH	liver function normalized with treatment, slight worsening on prednisolone weaning, normalized with azathioprine	[179]	
Erard et al.	80	F	10 days after 2nd dose	mRNA	Elevated bilirubin, AST, ALT, positive ANA, Elevated IgG	New-onset AIH	Steroids	Improved symptoms	[180]	3
Erard et al.	73	F	21 days after 1st dose	mRNA	Elevated bilirubin, AST,	New-onset AIH	Steroids	Improved symptoms	[180]	

					ALT, positive ANA, Elevated IgG					
Erard et al.	68	F	20 days after 1st dose	Adenoviral vector	Elevated bilirubin, AST, ALT, positive ANA, Elevated IgG, INR	New-onset AIH	None (died before treatment)	Death from liver failure and sepsis	[180]	
Fimiano et al.	63	F	7 weeks after 2nd dose	mRNA	Elevated AST, ALT, ALP, GGT, bilirubin, positive anti-thyroglobulin antibodies, Elevated IgG, highly Elevated anti-SARS-CoV2 spike protein antibody	New-onset AIH	Methylprednisolone, Azathioprine	Improved symptoms, reduction in transaminases and bilirubin	[181]	1
Camacho-Domínguez et al.	79	M	15 days after 1st dose	Adenoviral vector	Elevated bilirubin, AST, ALT, positive ASMA, positive ANA, Elevated IgG, highly Elevated anti-SARS-CoV-2 antibodies	New-onset AIH	Hydrocortisone, Prednisone, Azathioprine	Improved symptoms, reduction in liver enzymes and bilirubin	[182]	1
Ghielmetti et al.	63	M	7 days after 1st dose	mRNA	Elevated AST, ALT, GGT, ALP, bilirubin, positive ANA, positive anti-gastric parietal cells antibody, Elevated IgG	New-onset AIH	Prednisone	Improved symptoms, reduction in liver enzymes and bilirubin	[183]	1

Vuille-Lessardet al.	76	F	2-3 days after 1st dose	mRNA	Elevated bilirubin, AST, ALT, ALP, GGT, positive ANA, positive ASMA, positive anti-actin antibody, Elevated IgG	New-onset AIH	Prednisolone, Azathioprine	Improved symptoms, normalization of liver enzymes, no AIH relapse after stopping therapy	[184]	1
Suzuki et al.	80	F	10 days after 2nd dose	mRNA	Elevated AST, ALT, bilirubin, positive ANA, Elevated IgG	New-onset AIH	Prednisone	Improved symptoms, reduction in liver enzymes and bilirubin	[185]	3
Suzuki et al.	75	F	4 days after 2nd dose	mRNA	Elevated AST, ALT, bilirubin, positive ANA, Elevated IgG	New-onset AIH	Prednisone	Improved symptoms, reduction in liver enzymes and bilirubin	[185]	
Suzuki et al.	78	F	7 days after 1st dose	mRNA	Elevated AST, ALT, bilirubin, positive ANA, Elevated IgG	New-onset AIH	Prednisone	Improved symptoms, reduction in liver enzymes and bilirubin	[185]	
Palla et al.	40	F	1 month after completing vaccination	mRNA	Elevated transaminases, positive ANA, Elevated IgG	New-onset AIH	Prednisolone	Improved symptoms, normalization of	[186]	1

								transaminases		
Garrido et al.	65	F	2 weeks after 1st dose	mRNA	Elevated AST, ALT, GGT, bilirubin, positive ANA, Elevated IgG	New-onset AIH	Prednisolone	Improved symptoms, normalization of liver function tests and IgG levels	[187]	1
Avci et al.	61	F	2-3 days after 1st dose	mRNA	Elevated AST, ALT, GGT, ALP, bilirubin, positive ANA, positive ASMA, Elevated IgG	New-onset AIH	Prednisolone, Azathioprine	Improved symptoms, normalization of liver enzymes, resolved icterus	[188]	1
Cao et al.	57	F	2 weeks after 1st dose	CoronaVac	Elevated bilirubin, AST, ALT, ALP, GGT, positive ANA, positive anti–Sjögren syndrome antigen A and B, Elevated IgG	New-onset AIH	Ursodeoxycholic acid, Methylprednisolone, Azathioprine	Improved symptoms, no relapse during 5-month follow-up	[189]	1
Lodato et al.	43	F	2 weeks after 1st dose	mRNA	Elevated bilirubin, ALT, AST	New-onset AIH-like	N-acetyl-cysteine, Methylprednisolone	Improved symptoms, normalization of liver function tests	[190]	1
Zhou et al.	36	F	11 days after 1st dose	mRNA	Elevated ALT, AST, bilirubin, ANA, anti-	New-onset AIH	Prednisone, Azathioprine	Improved symptoms,	[191]	1

					double-stranded DNA, IgG			normalization of liver enzymes		
Zin et al.	47	M	3 days after 1st dose	mRNA	Elevated bilirubin, ALT, ALP, IgG, ANA	New-onset AIH	Prednisolone	Improved symptoms, normalization of liver enzymes	[192]	1
Rocco et al.	57	F	1 week after 2nd dose	mRNA	Elevated AST, ALT, bilirubin, ALP, GGT, IgG, and positive ANA antibody	New-onset AIH	Prednisone	Improvement with treatment	[193]	1
McShane et al.	71	F	4 days after vaccination	mRNA	Elevated bilirubin+ALP+ALT+AST, positive ANA, positive SMA, high IgG	New-onset AIH	Prednisolone	Improvement with treatment	[194]	1
Tan et al.	56	F	6 weeks after 1st dose	mRNA	Elevated ALT+AST+bilirubin+ALP, positive ANA+ASMA, high IgG	New-onset AIH	Budesonide	Improvement with treatment	[195]	1
Ghorbani et al.	62	M	3 days after 2nd dose	Inactivated	Elevated ALT+AST+ALP+bilirubin, negative ANA, ASMA, anti-LKM-1, and viral markers, histology showing lymphocyte/eosinophil	New-onset AIH	Ursodeoxycholic acid	Improvement without corticosteroids	[196]	1

					infiltrate, interface hepatitis, feathery change					
Londoño et al.	41	F	7 days after 2nd dose	mRNA	Elevated ALT, AST, bilirubin, GGT, ALP, positive ANA, ASMA, anti-SLA, anti-liver cytosol, high IgG	New-onset AIH	Prednisolone	Improvement with treatment	[197]	1
Izagirre et al.	47	F	24 days after 1st dose	Adenoviral vector	Elevated, ALT, AST, IgG, ANA, ASMA	New-onset AIH	Prednisone, Azathioprine	Normalization of transaminases in 3 months	[198]	5
Izagirre et al.	72	M	46 days after 2nd dose	mRNA	Elevated, ALT, AST, IgG, ANA	New-onset AIH	Prednisone, Azathioprine	Normalization of transaminases in 5 weeks	[198]	
Izagirre et al.	62	F	4 days after 2nd dose	Adenoviral vector	Elevated, ALT, AST, IgG, ANA	New-onset AIH	Prednisone, Azathioprine	Normalization of transaminases in 5 months	[198]	
Izagirre et al.	72	F	14 days after 2nd dose	mRNA	Elevated, ALT, AST, IgG, ANA	New-onset AIH	Prednisone	Normalization of transaminases in 5 months	[198]	
Izagirre et al.	59	F	9 days after 1st dose	mRNA	Elevated, ALT, AST, IgG, ANA	New-onset AIH	No treatment	Normalization of transaminases in 5 months	[198]	

Hasegawa et al.	82	F	7 days after 1st dose	mRNA	Elevated, AST, ALT, IgG, ANA, Total bilirubin	New-onset AIH with HCV history	Prednisolone	Discharged on hospital day 14	[199]	1

Table S6 Summary of case reports on T1DM after COVID-19 vaccination

Author	Age	Sex	Timeline of Symptom Onset	Type of Vaccine	Laboratory Features	Diagnosis	Treatment	Outcome	Ref	Case #
Sakurai et al.	36	F	3 days after 1st dose	mRNA	elevated glucose, β-hydroxybutyrate, acetoacetic acid, amylase, lipase, decreased serum C-peptide	New onset T1DM	Insulin	Improved	[203]	1
Tang et al.	50	M	6 days after 1st dose	Inactivated	elevated glucose, ketosis, metabolic acidosis, elevated HbA1c, elevated pancreatic enzymes, decreased serum C-peptide.	New onset T1DM	Insulin	Improved	[204]	1
Sato et al.	43	M	2 days after 2nd dose	mRNA	severe hyperglycemia, elecvated HbA1c, ketone bodies, low Fasting CPR	New onset T1DM	Insulin	Not well controlled	[205]	1

Tanaka et al.	60	M	2 days after 3rd dose	mRNA	hyponatremia, low serum cortisol, and low ACTH	New-onset T1DM	Hydrocortisone, insulin, S-1, oxaliplatin, nivolumab, capecitabine, nab-paclitaxel, ramucirumab	Died	[206]	1
Moon et al.	56	F	7 wks post 2nd dose	mRNA	Elevated HbA1c, FG; Positive for anti-GAD antibody	New-onset T1DM	Metformin, Glimepiride, Gemigliptin, Insulin	Improved	[207]	1
Yano et al.	51	F	28 days post 1st dose	mRNA	Hyperglycemia, Elevated HbA1c, Metabolic acidosis, Increased anion gap, Ketonemia, Exhaustion of endogenous insulin secretion, Positive insulin autoantibody, Autoimmunity against thyroid gland	New-onset T1DM	IV saline, IV insulin infusion, Subcutaneous insulin therapy (insulin glargine U-300, insulin lispro)	Improved	[208]	1

Bleve et al.	57	F	Few days after 1st dose	adenoviral vector	elevated FPG, HbA1c, Anti-GAD, Anti-IA2, Anti-TransGlut IgA	New-onset T1DM with T2DM history	Insulin	Improved	[209]	
Bleve et al.	61	F	Few days after 2nd dose	mRNA	elevated FPG, HbA1c, Anti-GAD, Anti-TPO, metabolic acidosis	New-onset T1DM	Insulin, corticosteroid	Improved	[209]	2
Sasaki et al.	73	F	4 weeks after 2nd dose	mRNA	elevated HbA1c, casual blood glucose, anti-GAD antibody, insulin autoantibody, decreased serum C-peptide (glucagon stimulation test)	New-onset T1DM	Insulin	Improved	[210]	1
Sasaki et al.	45	F	3 days after 1st dose	mRNA	elevated glucose, β-hydroxybutyrate, acetoacetate, hemoglobin	New onset T1DM	Insulin	Improved	[211]	1

					A1c, decreased serum C-peptide, metabolic acidosis					
Patrizio et al.	52	M	4 weeks after 2nd dose	mRNA	elevated HbA1c, hyperthyroidism markers (low TSH, elevated fT3, elevated fT4), positive TRAb, TgAb, TPOAb, positive GAD65Ab, low serum C-peptide	New-onset T1DM with T2DM history	Insulin, Methimazole, Atenolol	Improved	[212]	1
Aydoğan et al.	56	M	15 days after 2nd dose	mRNA	elevated FPG, HbA1c, positive GAD65Ab, ketonuria, lower limit fasting insulin and C-peptide	New onset T1DM	Basal-bolus insulin therapy	Improved	[213]	4
Aydoğan et al.	48	M	2 months after 2nd dose	mRNA	elevated FPG, HbA1c,	New onset T1DM	Medical nutrition therapy	Frequent hypoglycemia with	[213]	

					positive GAD65Ab			low-carb diet		
Aydoğan et al.	27	F	3 weeks after 2nd dose	mRNA	elevated HbA1c, FPG, low C-peptide, positive GAD65Ab	New onset T1DM	Basal-bolus insulin therapy, medical nutrition therapy	Improved	[213]	
Aydoğan et al.	36	M	15 days after 2nd dose	mRNA	elevated random plasma glucose, metabolic acidosis, ketonuria, low C-peptide, high GAD65Ab	New onset T1DM	Basal-bolus insulin therapy	Improved	[213]	
Kobayashi et al.	59	M	15 weeks after 2nd dose	mRNA	elevated serum glucose, blood ketone bodies, anion gap, HbA1c, severely acidotic, elevated pancreatic exocrine enzymes	New onset T1DM	Insulin	Improved	[214]	1
Ohuchi et al.	45	M	3 days after 2nd dose	mRNA	elevated blood sugar, Low C-peptide	New onset T1DM	Adjuvant treatment with nivolumab	NR	[215]	1

					Reactivity, reduced C-peptide					
Huang	39	F	6 days after 4th dose	Protein Subunit	elevated serum glucose, decreased bicarbonate and increased anion gap, low C-peptide.	New onset T1DM	Insulin	Improved	[216]	1

Table S7 Summary of case reports on MG after COVID-19 vaccination

Author	Age	Sex	Timeline of Symptom Onset	Type of Vaccine	Laboratory Features	Diagnosis	Treatment	Outcome	Ref	Case #
Huang et al.	53	M	1 day after 1st dose	Adenoviral vector	Elevated serum anti-AChR antibody titer. The repetitive stimulation test in the accessory nerve showed decremental change.	New-onset MG	pyridostigmine, prednisone	Improved	[220]	1
Watad et al.	72	M	1 day after 2nd dose	mRNA	EMG-decrement of 28–46% on facial and shoulder muscles	New-onset MG	plasma exchange, Prednisolone	Improved	[221]	2
Watad et al.	73	M	7 days after 2nd dose	mRNA	EMG- borderline decrement, markedly pathologic jitter	New-onset MG	Pyridostigmine, plasma exchange, Prednisolone	mechanical ventilation	[221]	
Poli et al.	65	M	3 days after 3rd dose	mRNA	Elevated anti-AChR antibody titers	New-onset ocular MG	corticosteroids and intravenous immunoglobulins, plasmapheresis	Improved	[222]	1
Chavez et al.	82	M	2 days after 2nd dose	mRNA	Elevated Ach receptor binding Ab, Ach receptor modulating Ab and striational Ab titer and an EMG showed decrement with repeated nerve stimulation.	New-onset MG	IV pyridostigmine, IVIG and steroids	Improved	[223]	1
Maher et al.	52	M	1 day after 2nd dose	Adenoviral vector	SFEMG test revealed a neuromuscular transmission defect and highly	New-onset ocular MG	pyridostigmine, prednisone	Partially improved	[224]	1

					suggestive of myasthenia gravis.					
Chaturvedi et al.	61	M	14 days after 2nd dose	Adenoviral vector	Positive neostigmine test and acetylcholine receptor antibodies	New-onset of ocular MG	pyridostigmine, prednisone, azathioprine	Fully Recovered	[225]	1
Fanella et al.	90	M	10 days after 2nd dose	mRNA	RNS revealed a postsynaptic neuromuscular junction disorder. Positive serum AChR antibody. Thyroid US showed multinodular struma.	New-onset MG	pyridostigmine	Improved	[226]	3
Fanella et al.	80	M	6 days after 2nd dose	mRNA	RNS revealed a postsynaptic neuromuscular junction disorder. Positive serum AChR antibody	New-onset MG	pyridostigmine, plasma exchange, azathioprine	Recovered	[226]	
Fanella et al.	55	M	28 days after 2nd dose	mRNA	RNS revealed a postsynaptic neuromuscular junction disorder. Positive Serum AChR antibody.	New-onset MG	pyridostigmine, IVIG, prednisone	Recovered	[226]	
Abicic et al.	65	M	21 days after 3rd dose	mRNA	Highly positive anti-nAChR antibodies	New-onset ocular MG	pyridostigmine, prednisone	Improved	[227]	1
Su et al.	39	F	7 days after 1st dose	mRNA	Negative anti-AChR antibody titer	New-onset ocular MG	pyridostigmine, intravenous immunoglobulins, prednisolone	Improved	[228]	1
Papadopoulou et al.	70	F	10 days after 2nd dose	mRNA	NR	New-onset MG	IVIG, plasma exchange, rituximab	Improved	[229]	1

Lee et al.	33	F	1 day after 2nd dose	mRNA	RNST revealed a significant decrement response in the right orbicularis oculi. Negative serum AchR antibody titer	New-onset MG	pyridostigmine	Partial improvement	[230]	1
Tavares-Júnior et al.	68	F	30 days after 4th dose	Adenoviral vector	Electroneuromyography with repetitive stimulation of the four limbs revealed a decrement pattern, consistent with myasthenia gravis.	New-onset MG	pyridostigmine, plasma exchange, azathioprine, and prednisone	Stable	[231]	1
Slavin et al.	60	M	6 days after 3rd dose	mRNA	Postexercise exhaustion with stimulation at the right facial nerve with more than 10% decrement of amplitude	New-onset MG	pyridostigmine	Improved	[232]	1
Croitoru et al.	78	M	15 days after 3rd dose	mRNA	Elevated serum levels of antibodies against acetylcholine receptors	New-onset MG	pyridostigmine, pyridostigmine, IVIG	Improved	[233]	1
Galassi et al.	73	M	8 days after 1st dose	Adenoviral vector	Positive serum titer of anti- AChR antibodies.	New-onset ocular MG	pyridostigmine	Improved	[234]	1
Ramdas et al.	13	F	14 days after 1st dose	mRNA	Seropostivity: AChR negative; RNS: positive	New-onset MG	Pyridostigmine, Prednisolone	NR	[235]	7
Ramdas et al.	59	M	2 days after 1st dose	Adenoviral vector	Seropostivity: AChR positive; RNS: No data	New-onset MG	Pyridostigmine, Prednisolone	NR	[235]	

Ramdas et al.	63	M	3 days after 3rd dose	mRNA	Seropostivity: AChR positive; RNS: No data	New-onset MG	Pyridostigmine	NR	[235]	
Ramdas et al.	73	M	12 days after 3rd dose	mRNA	Seropostivity: AChR positive; SfEMG: increasing jitter	New-onset MG	Pyridostigmine, IVIG, Prednisolone	NR	[235]	
Ramdas et al.	50	M	7 days after 1st dose	mRNA	Seropostivity: AChR positive; RNS: normal	New-onset MG	Pyridostigmine	NR	[235]	
Ramdas et al.	83	F	6 days after 1st dose	mRNA	Seropostivity: AChR positive; RNS: normal; SfEMG: not available	New-onset MG	Pyridostigmine, IVIG, Prednisolone	NR	[235]	
Ramdas et al.	77	M	3 days after 1st dose	Adenoviral vector	Seropostivity: AChR positive; RNS and SfEMG: positive	New-onset MG	Pyridostigmine, plasma exchange, Prednisolone, required intensive care support	NR	[235]	
Sansone et al.	64	F	12 days after 2nd dose	mRNA	de novo anti-acetylcholine receptor antibody positive	New-onset MG	NR	NR	[236]	1
Hoshina et al.	30	M	2 days after 2nd dose	mRNA	Borderline elevated anti-AChR antibody	New-onset MG	Pyridostigmine, prednisone	Improvement in symptoms but continued to fluctuate	[237]	1
Kang et al.	35	M	7 days after 1st dose	Adenoviral vector	Positive ANA, anti-AChR antibodies	New-onset ocular MG	NR	NR	[238]	1
Özenç et al.	28	F	3 weeks after first dose, worsened after second dose	mRNA	Positive neostigmine test, typical electromyographic findings	New-onset MG	IVIG and oral pyridostigmine	Recovered	[239]	1

Table S8 Summary of case reports on AA after COVID-19 vaccination

Author	Age	Sex	Timeline of Symptom Onset	Type of Vaccine	Clinical Features	Diagnosis	Treatment	Outcome	Ref	Case #
Lee et al.	80	M	1 week after 1st dose	mRNA	A pull test gave a positive result, and cadaveric and exclamation point hairs were noted upon trichoscopy	New-onset AA	Topical immunotherapy with squaric acid dibutylester combined with topical 5% minoxidil	No improvement	[243]	1
Martora et al.	7	F	20 days after 2nd dose	mRNA	NR	New-onset AA	NR	NR	[244]	1
Gamonal et al.	27	F	15 days after 3rd dose	mRNA	Trichoscopy showed yellow dots, black dots, dystrophic hair, and white hairs of repilation. The biopsy displayed a mild lymphocytic infiltrate around the outer follicular sheath without signs of fibrosis	SLE, AA	oral prednisolone and HCQ	NR	[245]	1
Abdalla et al.	63	F	2 weeks after 1st dose	mRNA	complete loss of scalp, facial, and body hair (presented with hypothyroidism, prediabetes and thalassemia trait)	New-onset AA	NR	referral to dermatologist	[246]	1
Scollan et al.	33	F	2 months after 2nd dose	mRNA	Extensive scalp alopecia with focal regrowth areas	New-onset AA	Tofacitinib citrate	Decreased hair loss, increased regrowth	[247]	6

Scollan et al.	29	F	Within 1 week after 2nd dose	mRNA	Two localized areas of scalp alopecia with regrowth	New-onset AA	ILTAC	Pending treatment outcome	[247]	
Scollan et al.	22	M	1 month after 2nd dose	mRNA	Scalp and beard alopecia, 30% scalp loss, 80% beard loss (Elevated thyroid antibody)	New-onset AA	Tofacitinib citrate	Pending treatment outcome	[247]	
Scollan et al.	15	M	Within 1 week after 2nd dose	mRNA	Two localized areas of scalp alopecia	New-onset AA	ILTAC	Pending treatment outcome	[247]	
Scollan et al.	61	M	2 weeks after 1st dose	mRNA	alopecia totalis	New-onset AA	Pending possible trial of oral tofacitinib citrate	Pending treatment outcome	[247]	
Scollan et al.	16	M	1-2 weeks after 1st dose	mRNA	Extensive scalp alopecia, sparse eyebrows and eyelashes	New-onset AA	Tofacitinib citrate	Pending treatment outcome	[247]	
Genco et al.	25	F	1 week	mRNA	Trichoscopy showed black dots; broken hair; yellow dots	New-onset AA	minoxidil, topical clobetasol, topical growth factors, and intralesional triamcinolone	Not improved, follow-up	[248]	
Genco et al.	23	F	2 weeks	mRNA	Trichoscopy showed black dots; broken hair; yellow dots	New-onset AA	minoxidil, topical clobetasol, topical growth factors, and intralesional triamcinolone	Not improved, follow-up	[248]	5
Genco et al.	32	F	2 weeks	mRNA	Trichoscopy showed black dots; broken hair; yellow dots	New-onset AA	minoxidil, topical clobetasol, topical growth factors, and intralesional triamcinolone	Not improved, follow-up	[248]	

Genco et al.	31	M	3 weeks	mRNA	Trichoscopy showed black dots; yellow dots	New-onset AA	minoxidil, topical clobetasol, topical growth factors, and intralesional triamcinolone	Not improved, follow-up	[248]	
Genco et al.	51	M	2 weeks	mRNA	Trichoscopy showed black dots	New-onset AA	minoxidil, topical clobetasol, topical growth factors, and intralesional triamcinolone	Not improved, follow-up	[248]	
Su et al.	42	M	3 weeks after 1st dose	Adenoviral vector	Dermoscopy demonstrated yellow dots, black dots, short vellus hairs, exclamation mark hairs, and tapering hairs	New-onset AA	ILTAC	NR	[249]	1
Gallo et al.	31	M	3 weeks after 2rd dose	mRNA	Trichoscopy showed yellow-dots, black dots, dystrophic hair, and vellus hairs in the center and periphery of the patches	New-onset AA	NR	NR	[250]	1
Ganjei et al.	23	F	1 week after 1st dose	Adenoviral vector	the biopsy revealed peribulbar lymphocyte infiltration with increased miniaturized hairs.	New-onset AA	Oral prednisolone, tofacitinib	NR	[251]	2
Ganjei et al.	26	F	2 weeks after 2nd dose	Adenoviral vector	NR	alopecia totalis	Oral prednisolone	NR	[251]	

Wu et al.	20	F	2 weeks after 2nd dose	Inactivated	The pull test was diffusely positive. Dermoscopy (FotoFinder bodystudio ATBM) examination revealed broken hairs, black dots, and some exclamationmark hairs	New-onset AA	A combination of topical and oral steroids	clinical follow-up	[252]	1
Ho et al.	51	F	3 days after 1st dose	Adenoviral vector	Dermatoscopy revealed broken hairs, yellow dots, and occasional exclamation mark hairs	New-onset AA	Clobetasol propionate ointment, triamcinolone acetonide, oral tofacitinib	sparse white regrowth areas and exclamation point hair loss areas	[253]	1
Bardazzi et al.	21	F	2 weeks after 1st dose	mRNA	Trichoscopy of all cases showed features of disease activity, including black dots, broken hairs, and exclamation mark hairs	New-onset AA	intralesional triamcinolone	Partial hair regrowth	[254]	
Matsuda et al.	37	F	22 days after 1st dose	mRNA	Trichoscopy showed tapering hairs, broken hairs, black dots, and increase in vacant follicular ostia	New-onset AA	topical betamethasone butyrate propionate lotion	mostly recovered leaving only one oval bald patch	[255]	1
Iwata et al.	40	F	1 week after 1st	mRNA	NR	New-onset AA	oral prednisone, excimer lamp, oral cepharanthin, monoammonium glycyrrhizinate,	improved a little but no significant change	[256]	1

							herbal medications			
Arroyo et al	51	F	3 days after 3rd dose	Adenoviral vector	NR	New-onset AA	Mesotherapy	Improved	[257]	4
Arroyo et al	34	M	10 days after 3rd dose	Adenoviral vector	NR	New-onset AA	Mesotherapy	Improved	[257]	
Arroyo et al	40	M	7 days after 3rd dose	Adenoviral vector	NR	New-onset AA	Mesotherapy	Improved	[257]	
Arroyo et al	59	M	17 days after 3rd dose	Adenoviral vector	NR	New-onset AA	Mesotherapy, pulses of dexamethasone, clobetasol propionate	Improved	[257]	
AlZahrani et al.	44	M	2 weeks after 1st dose	mRNA	NR	New-onset AA	Prednisone, betamethasone valerate	No improvement	[258]	1
Teng et al.	34	F	4 weeks after 2nd dose	Adenoviral vector	NR	New-onset AA	Oral prednisolone, PRP therapy	Recovered	[259]	1
Aristizabal et al.	33	F	One month after 2nd dose	Inactivated	biopsies revealed follicular miniaturization, a marked catagen/telogen shift, and a peribulbar, predominantly lymphocytic inflammatory infiltrateTrichoscopy revealed newly growing hairs with exclamation pointhairs, black dots, and broken hairs	New-onset AA	Intralesional and topical corticosteroids	lesions did not progress, andsigns of regrowth were noted	[260]	1

Lo et al.	61	F	1 week after 2nd dose	mRNA	biopsies revealed follicular miniaturization, a marked catagen/telogen shift, and a peribulbar, predominantly lymphocytic inflammatory infiltrate	New-onset AA	topical fluocinonide, minoxidil, tacrolimus, minoxidil, intralesional triamcinolone acetonide	fully recovered	[261]	1
Shakoei et al.	74	M	2 days after 2st dose	Inactivated	Inactivated	New-onset AA	NR	Intralesional Corticosteroid injection	[262]	2
Shakoei et al.	37	M	6 days after 1st dose	Inactivated	Inactivated	New-onset AA	NR	Intralesional Corticosteroid injection	[262]	

Table S9 Summary of case reports on APS after COVID-19 vaccination

Author	Age	Sex	Timeline of Symptom Onset	Type of Vaccine	Laboratory Features	Diagnosis	Treatment	Outcome	Ref	Case #
Molina-Rios et al.	42	F	2 weeks after 1st dose	mRNA	Elevated ESR and CRP	New onset SLE and secondary APS	sulfasalazine, anticoagulation treatment	Improvement of symptoms	[29]	1
Moreno-Torres et al.	27	F	36 hours after1st dose	mRNA	Positive for lupus anticoagulant, aCL, aB2GP-1, and anti-phosphatidyl-serine/prothrombin	New-onset APS (Catastrophic)	Low molecular weight heparin (LMWH) and methylprednisolone, HCQ	Discharged with no improvement in renal function	[269]	1
Jinno et al.	71	F	1 day after 1st dose	mRNA	Positive for lupus anticoagulant, anti-beta2-GP1 IgM/IgG, Cardiolipin IgM/IgG	New-onset APS (Catastrophic)	Methylprednisolone, warfarin	Improvement of symptoms	[270]	1
Bryan et al.	50	M	2 weeks after 2nd dose	mRNA	Positive lupus anticoagulant, superficial and deep dermal and subcutaneous thrombosis	New-onset APS	prednisolone, Warfarin	Improvement of symptoms	[271]	1
Siagian et al.	39	F	6 weeks after 1st dose	Inactivated	Positive for lupus anticoagulant	New-onset APS	Diagnostic catheterization, anticoagulation treatment	Improvement of symptoms	[272]	1

Table S10 Summary of case reports on AAV after COVID-19 vaccination

Author	Age	Sex	Timeline of Symptom Onset	Type of Vaccine	Laboratory Features	Diagnosis	Treatment	Outcome	Ref	Case #
Matsuda et al.	71	F	The day after the 2nd dose	mRNA	Elevated ESR and CRP, low albumin, mildly anemic Hb level, positive RF	New-onset AAV	Oral glucocorticoid	Improved	[25]	1
Suzuki et al.	72	M	After 2nd dose	mRNA	Acute kidney injury, elevated MPO-ANCA	New-onset MPO-AAV	Hemodialysis, high-dose prednisolone, IV rituximab	Improved renal function	[274]	1
Dourado et al.	26	F	two weeks after 1st dose	mRNA	Elevated ESR, CRP, leukocytosis, eosinophilia, elevated serum creatinine, microscopic hematuria, positive ANCA-PR3 antibodies	New-onset AAV	Intravenous methylprednisolone, oral prednisolone, rituximab, methotrexate	Improved	[275]	2
Dourado et al.	47	M	3 months after 2nd dose	mRNA	Elevated serum creatinine, prostate-specific antigen, ESR, CRP, leukocytosis, proteinuria, hematuria, positive ANCA-MPO	New-onset AAGN	Oral cefuroxime, six plasmapheresis sessions, intravenous cyclophosphamide, methylprednisolone, oral prednisolone, rituximab, currently on prednisolone.	Currently under regular hemodialysis.	[275]	

Campos et al.	75	F	15 days after 2nd dose	Adenoviral vector	Elevated temperature, elevated creatinine requiring dialysis, positive ANCA	New-onset AAGN, lupus nephritis	IV methylprednisolone, cyclophosphamide, renal replacement therapy	Died	[276]	1
Marouço et al.	62	M	2 weeks after 1st dose, 2 weeks after 2nd dose	mRNA	Acute kidney injury, elevated MPO-ANCA	New-onset MPO-AAV	IV methylprednisolone, prednisolone, rituximab	Remained dialysis dependent	[277]	1
Qaisar et al.	77	M	2 weeks after Pfizer booster	adenoviral vector (2 doses),mRNA vaccine booster	Elevated MPO-ANCA, CRP, thrombocytosis	New-onset AAV	IV methylprednisolone, rituximab	Ongoing rehabilitation for vasculitic neuropathy	[278]	1
Al-Yafeai et al.	62	F	1 month after 1st dose	mRNA	Positive PR3-ANCA, Microscopic hematuria	New-onset AAV	Rituximab, cyclophosphamide, IV immunoglobulin (IVIG), IV methylprednisolone, plasmapheresis, broad-spectrum antimicrobials (linezolid, meropenem, micafungin)	Improved hemolysis and pulmonary hemorrhage; neurological function unchanged, transferred for further rehabilitation and management	[279]	1

Kawamura et al.	71	F	1 week after 2rd dose	mRNA	Positive for MPO-ANCA	New-onset AAV	Immunosuppressive therapy	Improved	[280]	1
Uddin et al.	59	M	17 days after 2nd dose	mRNA	Positive for ANCA, pauci-immune glomerulonephritis on biopsy	New-onset ANCA-associated renal vasculitis	Rituximab, steroids	Improved	[281]	1
Villa et al.	63	M	7 days after 1st dose	Adenoviral vector	Positive pANCA	New-onset ANCA-associated renal vasculitis	steroids, cyclophosphamide	Improved	[282]	1
Zamoner et al.	58	F	5 days after 1st dose	Adenoviral vector	Positive for P-ANCA and MPO	New-onset ANCA-associated renal vasculitis	cyclophosphamide, azathioprine, prednisone taper	Improved	[283]	1
Gen et al.	82	F	20 days after 3rd dose	mRNA	High MPO-ANCA	New-onset MPO-ANCA-associated vasculitis	prednisolone	Improved	[284]	1
Ma et al.	70	F	4 hours after 1st dose	Inactivated	Positive for MPO, p-ANCA, ANA	New-onset AAGN	Glucocorticoids, cyclophosphamide, oral low-dose steroids	Improved	[285]	1
Yadav et al.	52	F	12 days after a single dose	Adenoviral vector	Increased C-reactive protein, decreased C3 complement level, normal C4 level, p-ANCA and c-ANCA positivity	New-onset AAGN	Antibiotics, NSAIDs, methylprednisolone, cyclophosphamide, blood transfusion	Discharged after 10 days of hospitalization	[286]	1

Kim et al.	72	F	After 3rd dose	mRNA	Elevated serum creatinine, positive ANCA titers, antibodies against MPO, normal immunoglobulins	New-onset AAGN	Initial therapy: Intravenous pulse steroid therapy (methylprednisolone sodium succinate 500 mg/day for 3 days); Plasmapheresis initiated due to rapid deterioration of renal function; Intravenous cyclophosphamide (2.5 mg/kg) followed by oral cyclophosphamide; Tapered oral steroids per PEXIVAS trial protocol	Improved	[287]	1
El Hasbani et al.	47	F	3 days after 1st dose	mRNA	Positive for proteins, significant hematuria, elevated serum creatinine, elevated CRP, positive anti-MPO	New-onset AAGN	Intravenous methylprednisolone; Prednisone thereafter; Azathioprine	Improved	[288]	1

Christodoulou et al.	72	F	15 days after 2nd dose	mRNA	Increased levels of MPO-ANCA, impaired renal function, proteinuria, microscopic hematuria, active urine sediment, chest CT findings	New-onset MPO-AAV	Steroids, cyclophosphamide, plasmapheresis	remission	[289]	1
Feghali et al.	58	M	4 days after 2nd dose	mRNA	Elevated serum creatinine, hematuria, sub-nephrotic proteinuria, elevated C-ANCA, anti-PR3, chest CT findings	New-onset AAGN	Plasma exchange, prednisone, cyclophosphamide, rituximab	Remission, improved renal function	[290]	1
Yoshino et al.	56	M	Three weeks after 2nd dose	mRNA	Elevated serum creatinine, CRP, WBC, urinary protein, urinary occult blood, elevated MPO-ANCA	New-onset AAV	Methylprednisolone, prednisolone, cyclophosphamide, methotrexate	No signs of recurrent vasculitis	[291]	1
Tonutti et al.	49	M	Two weeks after 2nd dose	mRNA	Elevated AST, ALT, CRP, hypoalbuminaemia, hypergammaglobulinaemia, rheumatoid factor, ANA, new-onset anemia,	New-onset AAV	Methylprednisolone, rituximab	Complete remission at 6-month follow-up	[292]	1

					positive c-ANCA, high anti-PR3 autoantibodies				
Anderegg et al.	81	M	After 1st dose	mRNA	Positive PR3, AKI, ANCA, microscopic haematuria, non-nephrotic range proteinuria, Crescentic glomerulonephritis	New-onset AAV	Cyc + PLEX + steroids	Improved	[293]	1
Arjun Sekar et al.	52	M	14 days after 2nd dose	mRNA	Positive PR3, AKI, macroscopic haematuria, non-nephrotic range proteinuria, Crescentic glomerulonephritis, high serum creatinine	New-onset AAV	Rtx (1dose) f/b Cyc + steroids	NR	[294]	1
Shakoor et al.	78	F	16 days after 1st dose	mRNA	Positive MPO, AKI, microscopic haematuria, non-nephrotic range proteinuria, leukocyturia, Crescentic glomerulonephritis, high serum creatinine	New-onset AAV	Rtx + Steroids	Improved	[295]	1

Dube et al.	29	F	16 days after 2nd dose	mRNA	Positive MPO, AKI, microscopic haematuria, non-nephrotic range proteinuria, Crescentic glomerulonephritis, normal serum creatinine	New-onset AAV	Rtx + Cyc + steroids	Improved	[296]	1
Takenaka et al.	75	F	4 days after 1st dose	mRNA	Positive MPO, NA	New-onset AAV	Steroids	Improved	[297]	1
Gupta et al.	23	M	14 days after 2nd dose	mRNA	Positive MPO, Positive Anti-GBM, Positive ANA, AKI, microscopic haematuria, non-nephrotic range proteinuria, Crescentic glomerulonephritis, high serum creatinine	New-onset AAV	NA	NR	[298]	1
Hakroush et al.	79	M	14 days after 2nd dose	mRNA	Positive MPO, Positive ANA, leukocyturia, microscopic haematuria, nephrotic range proteinuria, AKI, Pauci-immune glomerulonephritis with	New-onset AAV	Cyc + steroids	Improved	[299]	1

					myoglobin cast nephropathy, high serum creatinine					
Klomjit et al.	82	F	28 days after 2nd dose	mRNA	Positive MPO, AKI, haematuria, proteinuria, Crescentic glomerulonephritis, high serum creatinine	New-onset AAV	Rtx + steroids	Improved	[300]	1
Prema et al.	58	M	14 days after 2nd dose	Inactivated	Positive PR3, Positive Anti-GBM, haemoptysis, AKI, Crescentic glomerulonephritis, high serum creatinine	New-onset AAV	Cyc + PLEX + steroids	Improved	[298]	2
Prema et al.	45	M	12 days after 1st dose	Inactivated	Positive MPO, Positive ANA, haemoptysis, AKI, Crescentic glomerulonephritis, high serum creatinine	New-onset AAV	Cyc + PLEX + steroids	Improved	[301]	
Caza et al.	76	M	11 days after 2nd dose	mRNA	Positive ANCA, Positive ANA, AKI, haematuria, proteinuria, Crescentic glomerulonephritis, high serum creatinine	New-onset AAGN	Rtx + steroids	Not Improved	[302]	6
Caza et al.	81	F	2 days after 2nd dose	mRNA	Positive ANCA, Positive ANA,	New-onset AAGN	Rtx	Not Improved	[302]	

					AKI, haematuria, proteinuria, Crescentic glomerulonephritis, high serum creatinine					
Caza et al.	76	F	5 days after 1st dose	mRNA	Positive ANCA, Positive ANA, AKI, haematuria, proteinuria, Crescentic glomerulonephritis, high serum creatinine	New-onset AAGN	Rtx + steroids	Not Improved	[302]	
Caza et al.	71	F	14 days after 2nd dose	mRNA	Positive ANCA, Positive ANA, haematuria, proteinuria, Crescentic glomerulonephritis, normal serum creatinine	New-onset AAGN	Rtx + steroids	Not Improved	[302]	
Caza et al.	65	F	14 days after 2nd dose	mRNA	Positive ANCA, AKI, haematuria, proteinuria, Crescentic glomerulonephritis, high serum creatinine	New-onset AAGN	Cyc + steroids	Not Improved	[302]	
Caza et al.	79	F	21 days after 2nd dose	mRNA	Positive ANCA, haematuria, proteinuria, Crescentic glomerulonephr	New-onset AAGN	Rtx	Not Improved	[302]	

					itis, normal serum creatinine					
Davidovic et al.	54	F	35 after 1st dose	mRNA	Positive MPO, AKI, haematuria, proteinuria, Pauci-immune glomerulonephritis, high serum creatinine	New-onset AAV	Rtx + steroids	Improved	[303]	1
Obata et al.	84	M	14 days after 2nd dose	mRNA	Positive MPO, microscopic haematuria, non-nephrotic proteinuria, Pauci-immune glomerulonephritis, normal serum creatinine	New-onset AAV	Steroids	Improved	[304]	1
Chen et al.	70	F	7 days after 1st dose	mRNA	Positive MPO, AKI, macroscopic haematuria, nephrotic range proteinuria, Crescentic glomerulonephritis, high serum creatinine	New-onset AAV	Rtx + PLEX + steroids	Improved	[305]	1
